# The synovial microenvironment suppresses chondrocyte hypertrophy and promotes articular chondrocyte differentiation

**DOI:** 10.1038/s41536-022-00247-2

**Published:** 2022-09-16

**Authors:** Michael Chau, Zelong Dou, Marta Baroncelli, Ellie B. Landman, Ameya Bendre, Masaru Kanekiyo, Alexandra Gkourogianni, Kevin Barnes, Lars Ottosson, Ola Nilsson

**Affiliations:** 1 Division of Pediatric Endocrinology and Center for Molecular Medicine, Department of Women’s and Children’s Health, Karolinska Institutet and University Hospital, Stockholm, Sweden; 2 Program in Developmental Endocrinology and Genetics, Eunice Kennedy Shriver National Institute of Child Health and Human Development, National Institutes of Health, Bethesda, MD USA; 3 Vaccine Research Center, National Institute of Allergy and Infectious Diseases, National Institutes of Health, Bethesda, MD USA; 4 School of Medical Sciences and Department of Pediatrics, Örebro University and University Hospital, Örebro, Sweden

**Keywords:** Bone, Cell growth, Experimental models of disease

## Abstract

During the development of the appendicular skeleton, the cartilaginous templates undergo hypertrophic differentiation and remodels into bone, except for the cartilage most adjacent to joint cavities where hypertrophic differentiation and endochondral bone formation are prevented, and chondrocytes instead form articular cartilage. The mechanisms that prevent hypertrophic differentiation and endochondral bone formation of the articular cartilage have not been elucidated. To explore the role of the synovial microenvironment in chondrocyte differentiation, osteochondral allografts consisting of articular cartilage, epiphyseal bone, and growth plate cartilage from distal femoral epiphyses of inbred Lewis rats expressing enhanced green fluorescent protein from a ubiquitous promoter were transplanted either in inverted or original (control) orientation to matching sites in wildtype littermates, thereby allowing for tracing of transplanted cells and their progenies. We found that no hypertrophic differentiation occurred in the growth plate cartilage ectopically placed at the joint surface. Instead, the transplanted growth plate cartilage, with time, remodeled into articular cartilage. This finding suggests that the microenvironment at the articular surface inhibits hypertrophic differentiation and supports articular cartilage formation. To explore this hypothesis, rat chondrocyte pellets were cultured with and without synoviocyte-conditioned media. Consistent with the hypothesis, hypertrophic differentiation was inhibited and expression of the articular surface marker lubricin (*Prg4*) was dramatically induced when chondrocyte pellets were exposed to synovium- or synoviocyte-conditioned media, but not to chondrocyte- or osteoblast-conditioned media. Taken together, we present evidence for a novel mechanism by which synoviocytes, through the secretion of a factor or factors, act directly on chondrocytes to inhibit hypertrophic differentiation and endochondral bone formation and promote articular cartilage formation. This mechanism may have important implications for articular cartilage development, maintenance, and regeneration.

## Introduction

The development of endochondral bones occurs through the condensation of mesenchymal cells at sites of future skeletal elements. Under the control of transcription factor *Sox9*, mesenchymal cells differentiate into chondrocytes that proliferate and produce a matrix rich in collagen type 2 (*Col2a1*) and proteoglycans^1^. At the center of these cartilaginous skeletal templates, the chondrocytes undergo hypertrophic differentiation during which they downregulate *Col2a1* expression, upregulate collagen type X (*Col10a1*) expression, and produce alkaline phosphatase necessary for matrix mineralization and vascular endothelial growth factor that attracts invading blood vessels and bone cells^1,2^ that form and expand the primary ossification center. Later, a secondary ossification center forms in the epiphysis that physically separates articular cartilage from the cartilaginous growth plate^3^.

Growth plate and articular cartilage have similar structures composed of distinct chondrocyte layers but differ substantially with respect to their function and fate. Growth plate cartilage is responsible for elongation of the appendicular skeleton and is entirely replaced by bone at the end of puberty^4–7^, whereas articular cartilage provides a low friction surface that protects the ends of long bones and persists throughout life given the absence of degenerative articular cartilage disease or trauma^8–10^. Despite their common origin, articular and growth plate chondrocytes are often considered to be separate cell lineages. However, the key cellular and molecular mechanisms responsible for their divergent differentiation are currently unknown^11^.

We hypothesized that the lack of hypertrophic differentiation at the articular surface is due to one or several factors in the joint microenvironment and/or that the epiphyseal or metaphyseal bone compartments promote endochondral ossification. To address this hypothesis in vivo, we switched the microenvironments of articular and growth plate cartilage by transplanting distal femoral osteochondral allografts containing articular cartilage, epiphyseal bone, and growth plate cartilage in inverted or original (control) orientation. To allow tracing of transplanted cells and their progenies, allografts were harvested from enhanced green fluorescent protein (eGFP)-positive rats and transplanted into wildtype littermates. We next cultured rat chondrocyte pellets with and without different types of cell-conditioned media and found that synoviocyte-conditioned media inhibited hypertrophic differentiation and induced expression of the articular surface marker lubricin (*Prg4*). Lastly, the physical nature of the putative synoviocyte factor(s) was analyzed.

## Results

### Osteochondral allograft transplantation and cell tracing

**Fig. 1:**
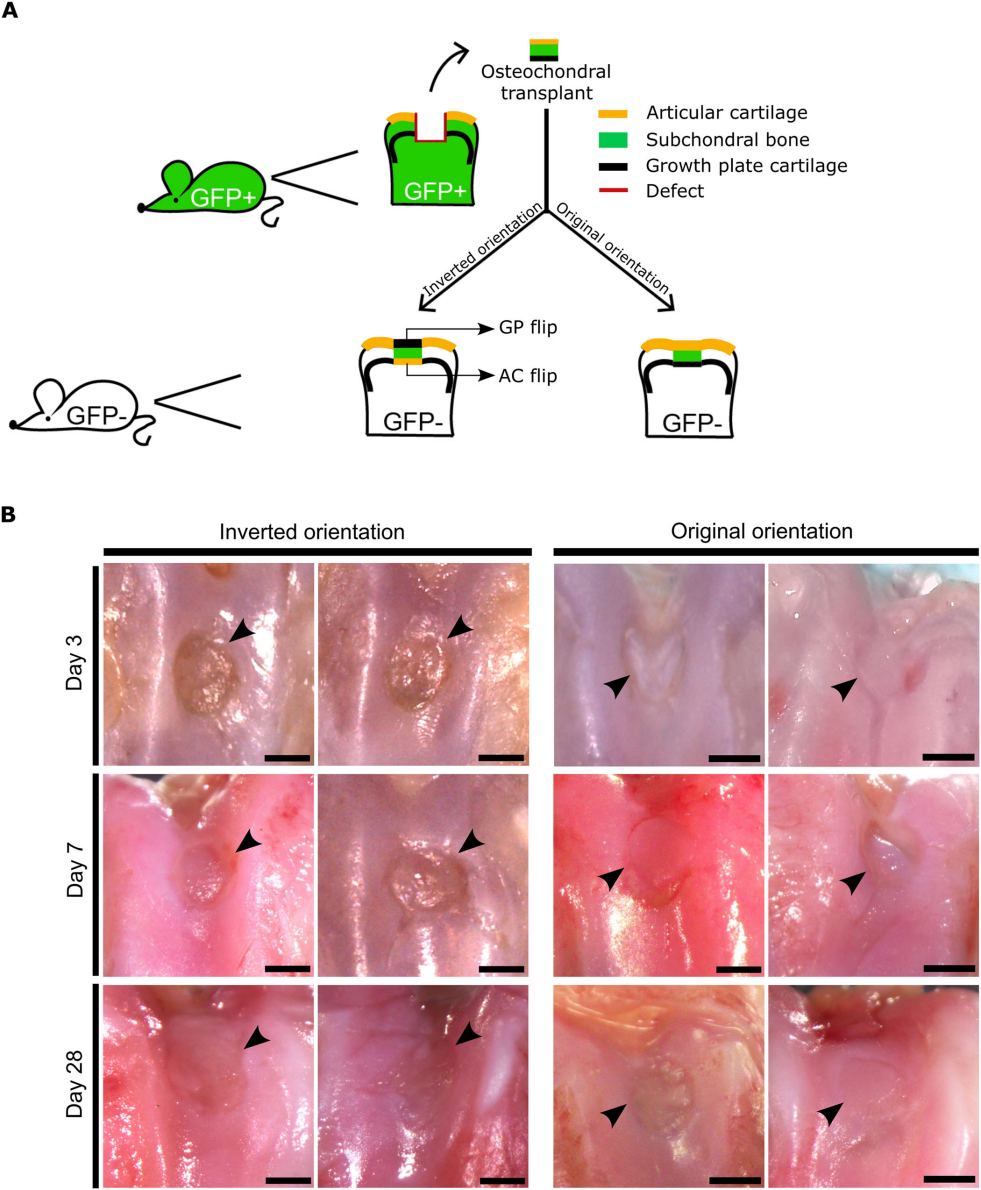
Experimental design of transplantation surgery and distal femoral articular surfaces at different postoperative timepoints. Osteochondral allografts consisting of articular cartilage, epiphyseal bone, and growth plate cartilage were harvested from distal femurs of inbred GFP-expressing rats and transplanted into matching sites of wildtype (GFP-negative) littermates in inverted (flip) or original (control) orientation (**A**). Donor and recipient animals were 4 weeks of age on the day of surgery. Photographs of intercondylar articular surfaces at postoperative day 3, 7, and 28, respectively (**B**). Black arrowheads indicate the transplanted allografts. Scale bar: 1000 μm. GP flip: the growth plate transplanted to the articular surface. AC flip: the articular cartilage is transplanted deep into the epiphysis.

**Fig. 2:**
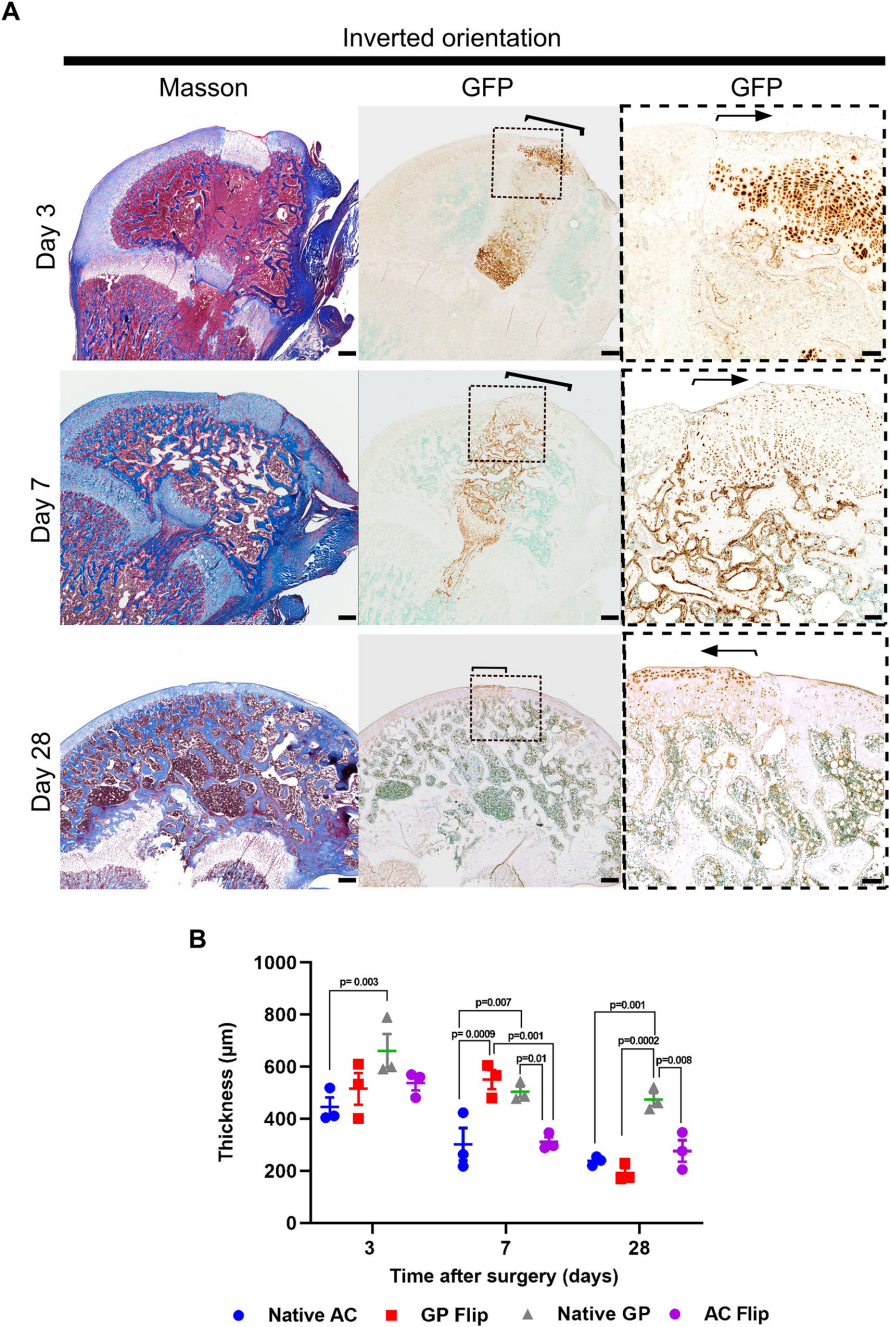
Growth plate cartilage transplanted to the articular surface gradually remodeled into articular-like cartilage. Osteochondral allografts consisting of articular cartilage, epiphyseal bone, and growth plate cartilage were harvested from distal femurs of inbred GFP-expressing rats and transplanted into matching sites of wildtype (GFP-negative) littermates in inverted orientation. **A** Consecutive sections of formalin-fixed, decalcified, and paraffin-embedded distal femoral epiphyses from GFP-negative recipient rats at postoperative day 3, 7, and 28 were stained with Masson’s trichrome or GFP immunohistochemistry to localize cells derived from the transplants. Arrows and brackets delineate osteochondral allografts. Scale bar: 500 μm and 100 μm in low and high-power images, respectively. **B** Thickness of growth plate cartilage transplanted to the articular surface (GP flip), articular cartilage transplanted to the epiphysis (AC flip), recipient’s own articular cartilage (native AC), and recipient’s own growth plate cartilage (native GP) was assessed (*n* = 3 per timepoint, 10 measurements per sample, two-way ANOVA followed by Tukey’s test). Bars represent average ± SEM. Each data point represents one animal.

**Fig. 3:**
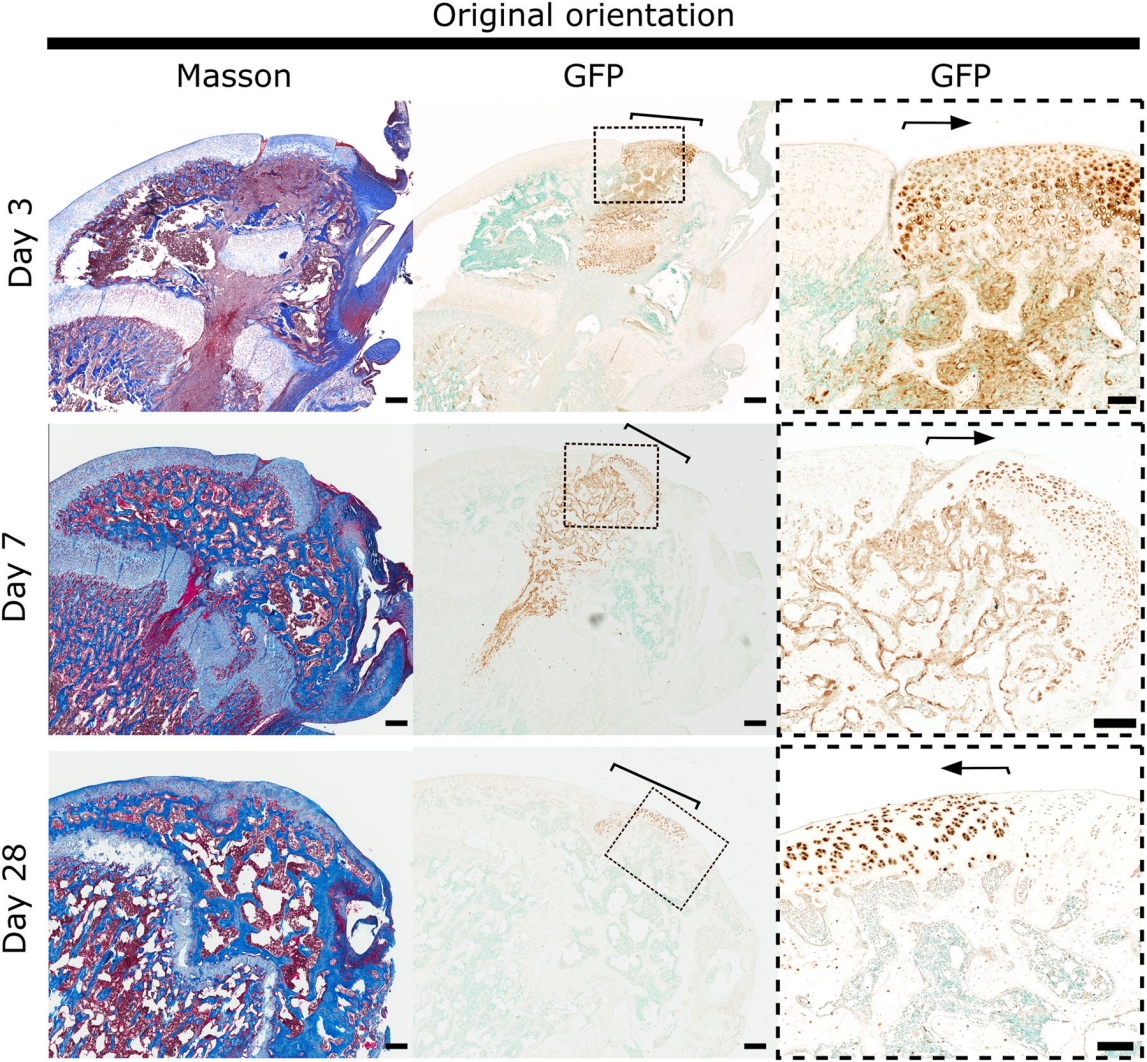
Articular cartilage transplanted to the articular surface remained intact. Osteochondral allografts consisting of articular cartilage, epiphyseal bone, and growth plate cartilage were harvested from distal femurs of inbred GFP-expressing rats and transplanted to matching sites of wildtype (GFP-negative) littermates in original orientation (control). Consecutive sections of formalin-fixed, decalcified, and paraffin-embedded distal femoral epiphyses from GFP-negative recipient rats at postoperative day 3, 7, and 28 were stained with Masson’s trichrome or with GFP immunohistochemistry to localize transplanted cells and their derivatives and displayed in low (Masson’s Trichrome) or low and high power (GFP immunohistochemistry). Brackets and arrows delineate osteochondral allografts at the joint surface. Note that at 28 days the growth plate injury and proximal end of allograft are not visible on the imaged tissue section. Scale bar: 500 μm and 100 μm in low and high-power images, respectively.

**Fig. 4:**
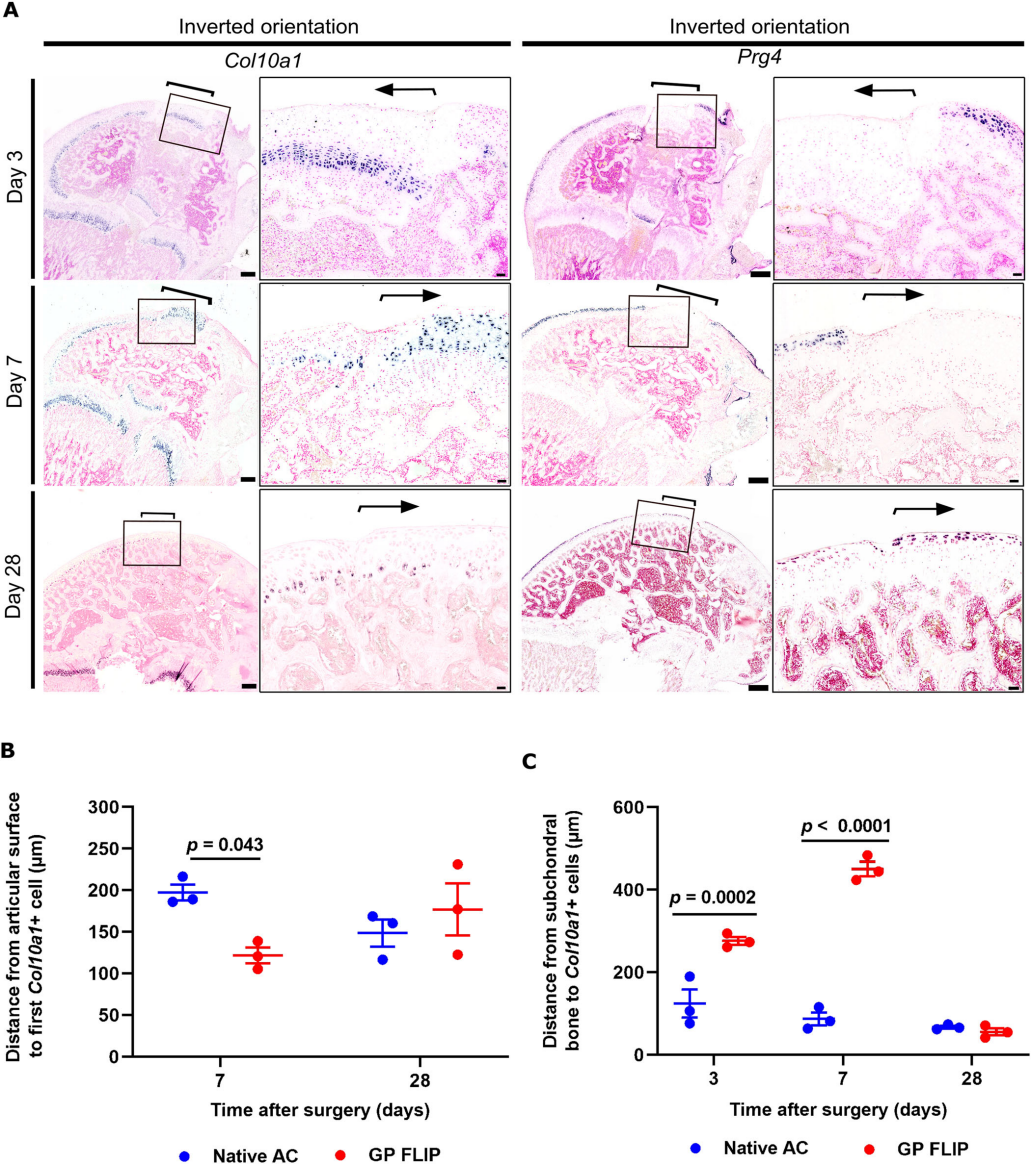
Localization of Col10a1 and Prg4 expression in transplants inserted in inverted orientation. Osteochondral allografts were harvested from distal femurs of inbred GFP-expressing rats and transplanted to matching sites of wildtype (GFP-negative) littermates in inverted orientation and localized by GFP immunohistochemistry. **A** Hypertrophic and superficial zone chondrocyte differentiation was assessed by Col10a1 and Prg4 in situ hybridization (purple coloration) on consecutive distal femoral sections of recipient rats at postoperative day 3, 7, and 28. Brackets and arrows delineate the location of the transplanted osteochondral allografts. **B** Distance from articular cartilage surface to first Col10a1-positive cells as well as (**C**) distance from subchondral bone to Col10a1-positive cells were assessed in growth plate transplanted to the articular surface (GP flip) and recipients’ articular cartilage (native AC) (*n* = 3 per timepoint; **B**: 10–12 measurements per sample; **C**: 30 measurements per sample; **B**, **C** Two-way ANOVA followed by Tukey’s test). Scale bar: 500 μm and 50 μm in low- and high-power images, respectively. Bars represent average ± SEM. Each datapoint represents an animal.

**Fig. 5:**
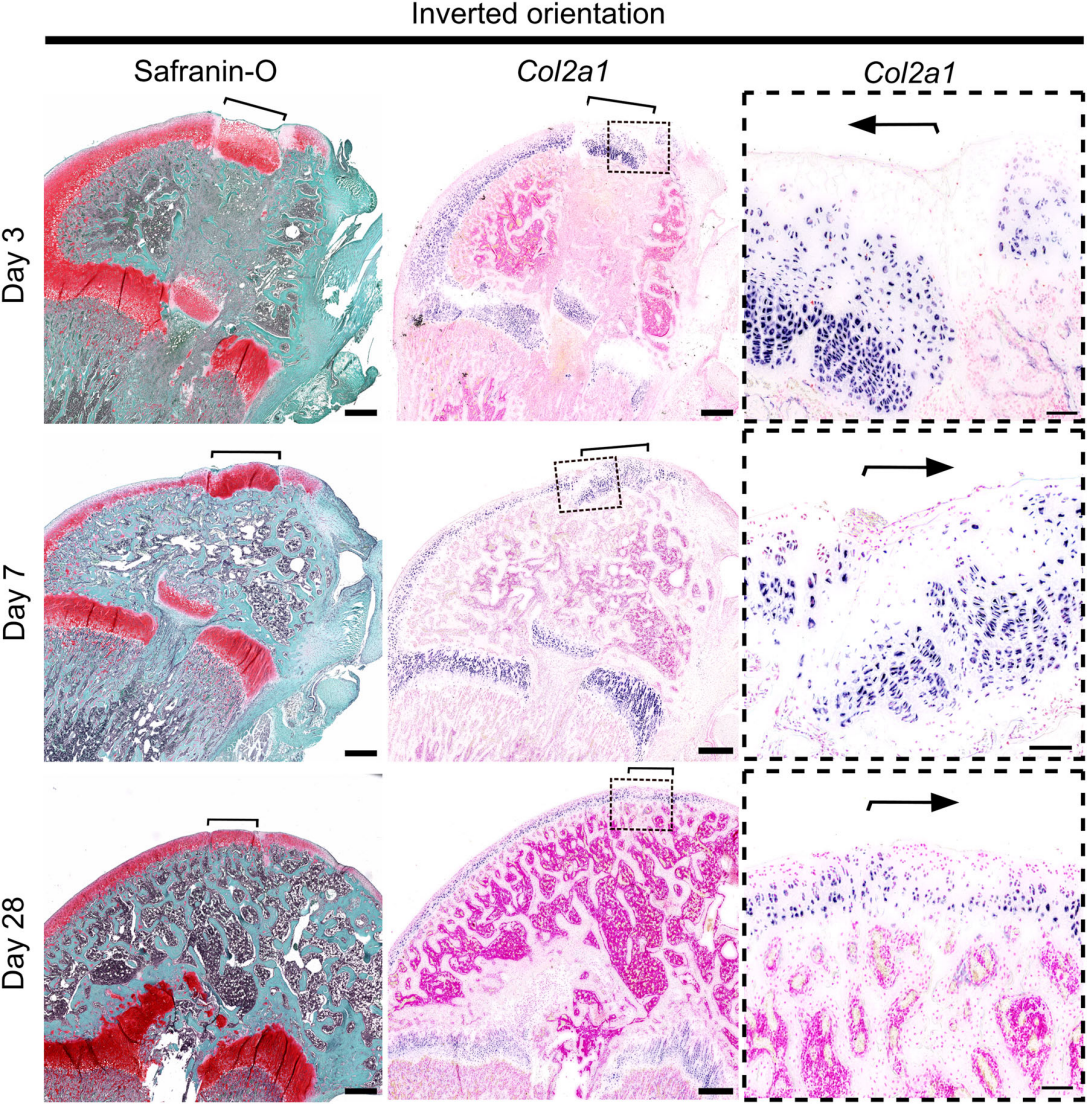
Proteoglycan content and Col2a1 expression were maintained in transplants inserted in inverted orientation. Osteochondral allografts consisting of articular cartilage, epiphyseal bone, and growth plate cartilage from GFP-expressing rats were transplanted into matching sites in wildtype (GFP-negative) littermate rats in inverted orientation. Proteoglycan content was visualized by Safranin-O-staining (red) in sections of distal femur obtained from transplant recipient rats on postoperative day 3, 7, and 28 and displayed in low power. Expression of chondrocyte marker Col2a1 mRNA was localized by non-radioactive digoxigenin in situ hybridization and displayed in low and high power. Brackets and arrows indicate the location of the transplanted osteochondral allografts. Scale bar: 500 μm in low and 100 μm in high-power images.

**Fig. 6:**
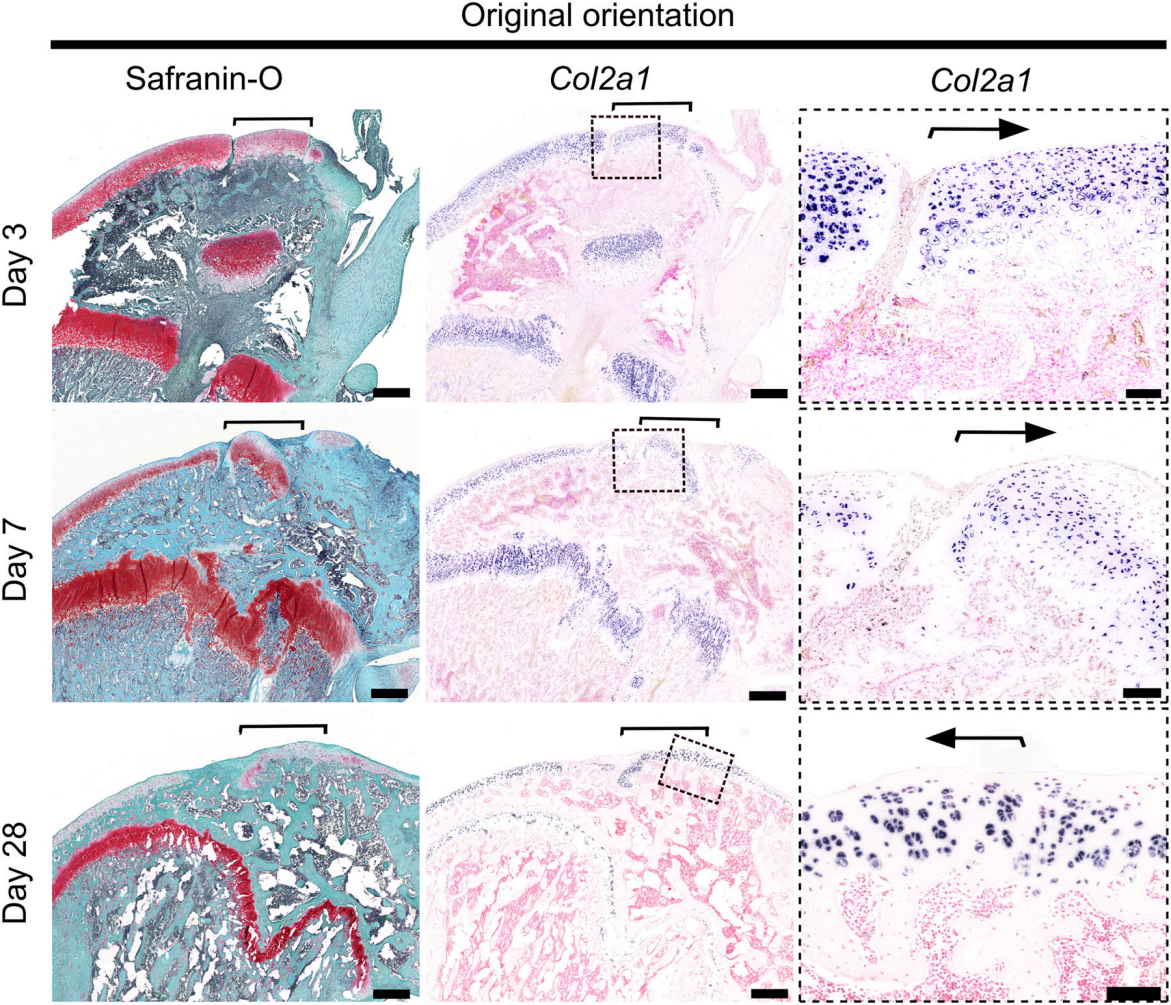
Proteoglycan content and Col2a1 expression were maintained in transplants inserted in original orientation. Osteochondral allografts consisting of articular cartilage, epiphyseal bone, and growth plate cartilage from distal femurs of inbred GFP-expressing rats were transplanted to matching sites in wildtype (GFP-negative) littermates in original orientation (control). Proteoglycan content was visualized by Safranin-O staining (red) in sections of distal femur in low power, and Col2a1 expression by non-radioactive digoxigenin in situ hybridization at postoperative day 3, 7, and 28 and visualized in low and high power. Brackets and arrows indicate the location of the transplanted allografts. Scale bar: 100 μm.

Recipient animals recovered rapidly after the surgeries and were able to ambulate on their hind legs immediately after recovery from anesthesia. None of the animals developed postsurgical infection or any signs of allograft rejection. One animal was noted to have medial luxation of the patella at the time of sacrifice. Macro- and microscopically transplanted osteochondral allografts healed in well and appeared to be vital at all postsurgical timepoints (Figs. 1B, 2A). The use of donors with ubiquitous eGFP expression (Lew-Tg(CAG-EGFP)YsRrrc) and *eGFP*-negative recipients (LEW/SsNHsd) enabled tracing of transplanted cells at all postsurgical timepoints by GFP immunohistochemistry (Figs. 2, 3). The osteochondral biopsies had been obtained from 4-week-old *eGFP*-positive donor rats (Fig. 1A). At this age the distal femoral growth plates are approximately 50% thicker than the articular cartilage. However, during the process of harvest and implantation the growth plates transplanted to the articular surface (GP flip) were compressed and also lost some hypertrophic cartilage, and therefore were about as thick as the surrounding recipient articular cartilage (native AC) but thinner than the recipient growth plate (Native GP) at 3 days post surgery (Fig. 2, *P* < 0.05, GP flip *vs*. native GP). At 7 days post surgery, the transplanted growth plates had regained their heights and were similar to those of the recipients’ growth plates (Fig. 2B; *P* = 0.724, GP flip *vs*. native GP), and were approximately 50% thicker than the surrounding recipient articular cartilage (Fig. 2B, *P* < 0.01, GP flip *vs*. native AC; *P* < 0.05, Native GP *vs*. native AC), likely due to continued growth plate chondrogenesis with active proliferation (Supplementary figure 1A) and hypertrophy (Fig. 4A). Thus, donor growth plate cartilage transplanted to the articular surface extended below recipient articular cartilage at postsurgery day 7 (Fig. 2A). At day 28 post surgery, however, GP flip had remodeled into an articular cartilage-like tissue with a thickness that was similar to that of the surrounding recipient native AC (Fig. 2B, GP flip: 229 ± 31 μm; native AC: 238 ± 10 μm, *P* = 0.987 GP flip *vs*. native AC) and thinner than that of recipient growth plate (Fig. 2B, native GP: 474 ± 25 μm, *P* < 0.01 for both native AC vs. native GP and GP flip *vs*. native GP). Additionally, from post surgery day 3 to 28, the osteochondral transplants gradually integrated with the recipient tissue as shown by the histochemical stainings (Figs. 2A, 3, 5, and 6). In order to explore whether apoptosis was an important mechanism in the remodeling of the transplanted biopsies, the percent TUNEL positive cells were assessed in the biopsy, near the biopsies and distant from the biopsies. We found that the fractions of TUNEL positive cells were relatively low in cartilage and also that apoptosis in the transplanted biopsies were similar to the surrounding tissues (Supplementary figure 4).

### Growth plate cartilage ectopically transplanted to the articular surface remodels into articular-like cartilage

Articular and growth plate cartilage placed in their original microenvironment maintained their respective histological structure and expression patterns of chondrocyte markers *Prg4*, *Col10a1, Col2a1* at all timepoints (Figs. 3, 6, supplementary figure 2). Interestingly, growth plate cartilage transplanted in inverted orientation at the articular surface with the hypertrophic zone cartilage facing the synovial joint and the resting zone towards epiphysis (GP flip) was gradually remodeled into an articular cartilage-like structure (Figs. 1B, 2A, 4, and 5). At postoperative day 3, most of the hypertrophic lacunas were empty (Fig. 2A), and no hypertrophic chondrocytes were detected close to the articular surface of the GP flip (Fig. 4A). At postoperative day 7, the most superficial cell layers of the GP flip consisted of small, actively proliferating (Supplementary figure 1A) chondrocytes that expressed *Col2a1* (Fig. 5) and eGFP (Fig. 2A), but not *Col10a1* or *Prg4* (Fig. 4A). Large, *Col10a1*-positive hypertrophic chondrocytes were detected beneath the superficial cell layers (Fig. 4B, *P* < 0.05 by analysis of variance (ANOVA); Fig. 4C, *P* < 0.0001 by ANOVA), indicating that the synovial joint microenvironment inhibits hypertrophic differentiation and might even cause de-differentiated of hypertrophic chondrocytes. Deeper into the allograft, underneath the layer of hypertrophic chondrocytes, proliferative zone and resting zone chondrocytes were still identifiable by their characteristic histological appearance (Figs. 2A, 5). Taken together, these observations may suggest that the synovial joint microenvironment inhibits hypertrophic differentiation and might even be able to cause de-differentiation of hypertrophic chondrocytes. Alternatively, hypertrophic chondrocytes from the original transplant may have been lost over time and the most superficial cells of the transplant detected on day 28 may have derived from proliferating chondrocytes.

At 28 days post surgery, growth plate cartilage transplanted to the articular surface (GP flip) had remodeled into a structure similar to the surrounding articular cartilage (native AC) but could still be readily detected by GFP immunohistochemistry (Fig. 2A). At this timepoint, no *Col10a1*-positive chondrocytes were detected close to the surface of the GP flips (Fig. 4A, B, *P* = 0.02 by ANOVA for GP flip *vs*. native AC). Instead, the superficial chondrocytes of GP flips were oriented parallel to the articular surface and expressed *Prg4* in a similar pattern and intensity as the superficial chondrocytes of the surrounding native AC (Fig. 4A) and the control transplant inserted in original orientation (Supplementary figure 2). These findings suggest that the synovial joint microenvironment not only inhibits hypertrophic differentiation, but also promotes differentiation into a superficial zone chondrocyte phenotype. Furthermore, resting zone chondrocytes and proliferative columns of the transplanted growth plates present on postoperative day 3 and 7 could no longer be detected on postoperative day 28 (Figs. 2A, 5). Instead, *Col10a1*-expressing hypertrophic chondrocytes were localized in the deep zone next

to the articular cartilage-epiphyseal bone interphase at the same level as in the adjacent native AC (Fig. 4A). Consistent with this observation, histomorphometry measurements of the location of *Col10a1*-positive cells in growth plate transplanted to the articular surface showed that *Col10a1*-positive cells were located close to the surface (Fig. 4B, GP flip vs. native AC, day 7, *P* = 0.02 by ANOVA) and far from the secondary ossification center during early timepoints (Fig. 4C, *P* < 0.001 and *P* < 0.0001, GP flip *vs*. native AC, day 3 and 7, respectively), but then were similar at postoperative day 28.

These findings suggest that the synovial joint microenvironment inhibits chondrocytes from hypertrophic differentiation at the articular surface and supports differentiation toward an articular chondrocyte-like phenotype.

### Articular chondrocytes placed ectopically at the level of the growth plate appear to migrate out of the articular cartilage

**Fig. 7:**
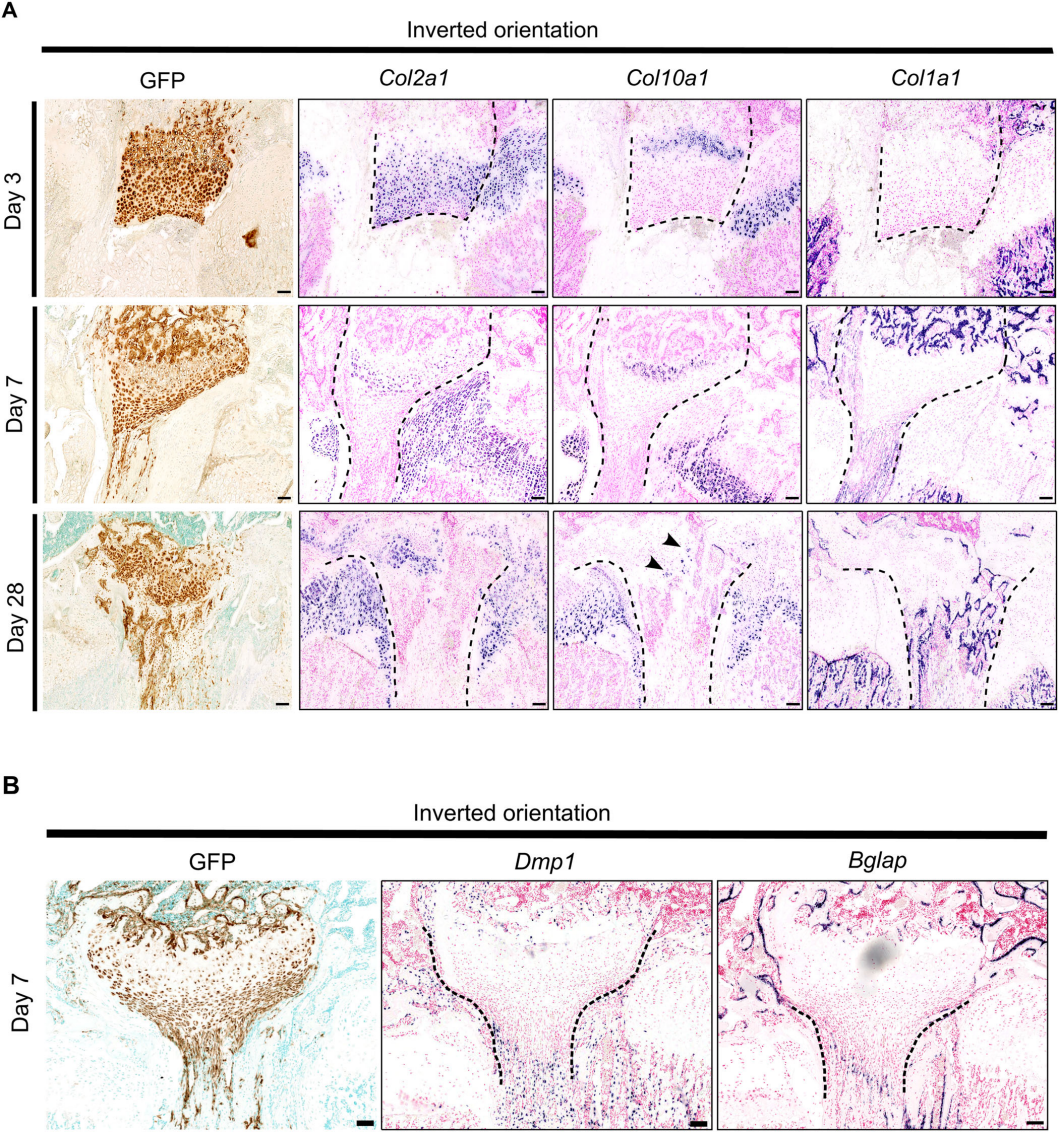
Tracing and differentiation of articular cartilage chondrocytes inserted ectopically at the level of the growth plate. Transplanted cells and their derivatives were localized by GFP immunohistochemistry staining (brown coloration) on sections of recipient distal femurs at postoperative day 3, 7, and 28 (**A**) and day 7 (**B**). Differentiation of transplanted chondrocytes was assessed by Col2a1, Col1a1, Col10a1, Prg4 (**A**) as well as Dmp1 and Bglap (**B**) in situ hybridizations (purple coloration) on consecutive sections. The microphotographs display the articular cartilage side of the transplants that had been ectopically placed at the location of the growth plate (AC flip). Dashed lines delineate allografts, based on localization by GFP immunohistochemistry. Scale bar: 100 μm.

Articular cartilage transplanted to the articular surface (control) exhibited a histological structure and marker expression pattern similar to the surrounding recipient articular cartilage (Figs. 3, 6, Supplementary figure 2), with Prg4-positive superficial chondrocytes at the surface and *Col10a1*-positive hypertrophic chondrocytes close to the subchondral bone (Supplementary figure 2) and integrated well with the surrounding recipient cartilage. Articular cartilage placed ectopically at the growth plate (AC flip) did not integrate with the recipient growth plate at any timepoint. At postoperative day 3, AC flip maintained an articular cartilage-like structure and expression pattern (Figs. 3, 6, Supplementary figure 2). However, on postoperative day 7, eGFP-positive cells appeared to be migrating out of the superficial zone. These cells were type I collagen (*Col1a1*)-positive and negative for Col2a1 and Col10a1 (Fig. 7A). Many cells were positive for *Dmp1* and *Bglap* (Fig. 7B), which might suggest that some articular chondrocytes transdifferentiated into the osteoblast lineage. However, at postoperative day 28 hypertrophic chondrocytes were detected at the position of the former superficial layer (Fig. 7A), which might suggest that some articular chondrocytes undergo hypertrophic differentiation before transdifferentiating into osteoblasts. These observations are largely consistent with the hypothesis that the synovial microenvironment act to induce and maintain the superficial chondrocyte phenotype.

### Synoviocytes produce a soluble factor that inhibits hypertrophic differentiation and induces articular cartilage superficial zone differentiation

**Fig. 8:**
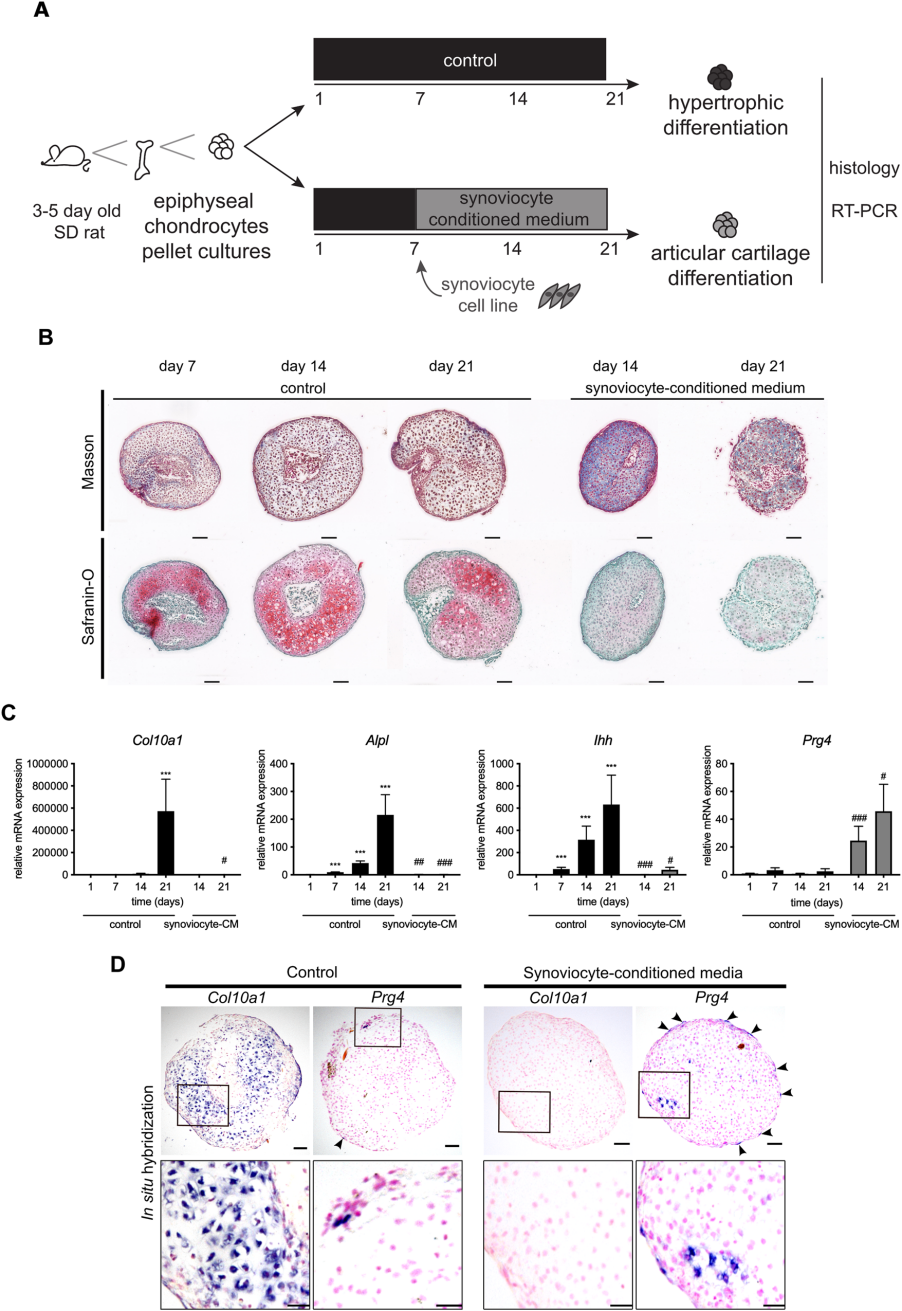
Synovium-conditioned medium contains factor(s) that inhibits hypertrophy and induce Prg4 expression in epiphyseal chondrocytes. **A** Schematic representation of chondrocyte pellet culture experiments. Epiphyses were dissected from 3–5-day-old rats and chondrocytes released by collagenase digestion, expanded, pelleted, and cultured for 7 days in regular chondrogenic media and then either exposed to conditioned media (CM; experimental condition) or chondrogenic medium (control) for another 7 (day 14) or 14 (day 21) days before subjected to histological or gene expression analyses. **B** Masson’s Trichrome staining and Safranin-O/Fast green staining were performed on sections of pellets cultured for 7, 14 (control & synoviocyte-conditioned media), or 21 (control & synoviocyte-conditioned media) days. **C** Gene expression of hypertrophic markers Col10a1, Alpl, and Ihh, and articular cartilage superficial layer marker Prg4 relative to 18 S rRNA were assessed by real-time PCR in pellets cultured for 1, 7, 14, and 21 days (n = 6 per timepoint and condition; one-way ANOVA followed by Dunnett’s method for pair-wise comparisons; ***P < 0.001 vs. control day 1; ^#^*P* < 0.05, ^##^P < 0.01, ^###^*P* < 0.001 vs. respective day of control culture (14 or 21 days)). Note that hypertrophic markers gradually increased, whereas Prg4 expression remained low when pellets were cultured in regular chondrogenic media. In contrast, hypertrophic markers remained low and Prg4 was induced when pellets were exposed to synoviocyte-CM. **D** In situ hybridization for Col10a1 and Prg4 were performed on sections of pellets cultured for 14 days. Scale bars represent 100 μm in low- (**B**, **D**) and 50 μm in high-power images.

**Fig. 9:**
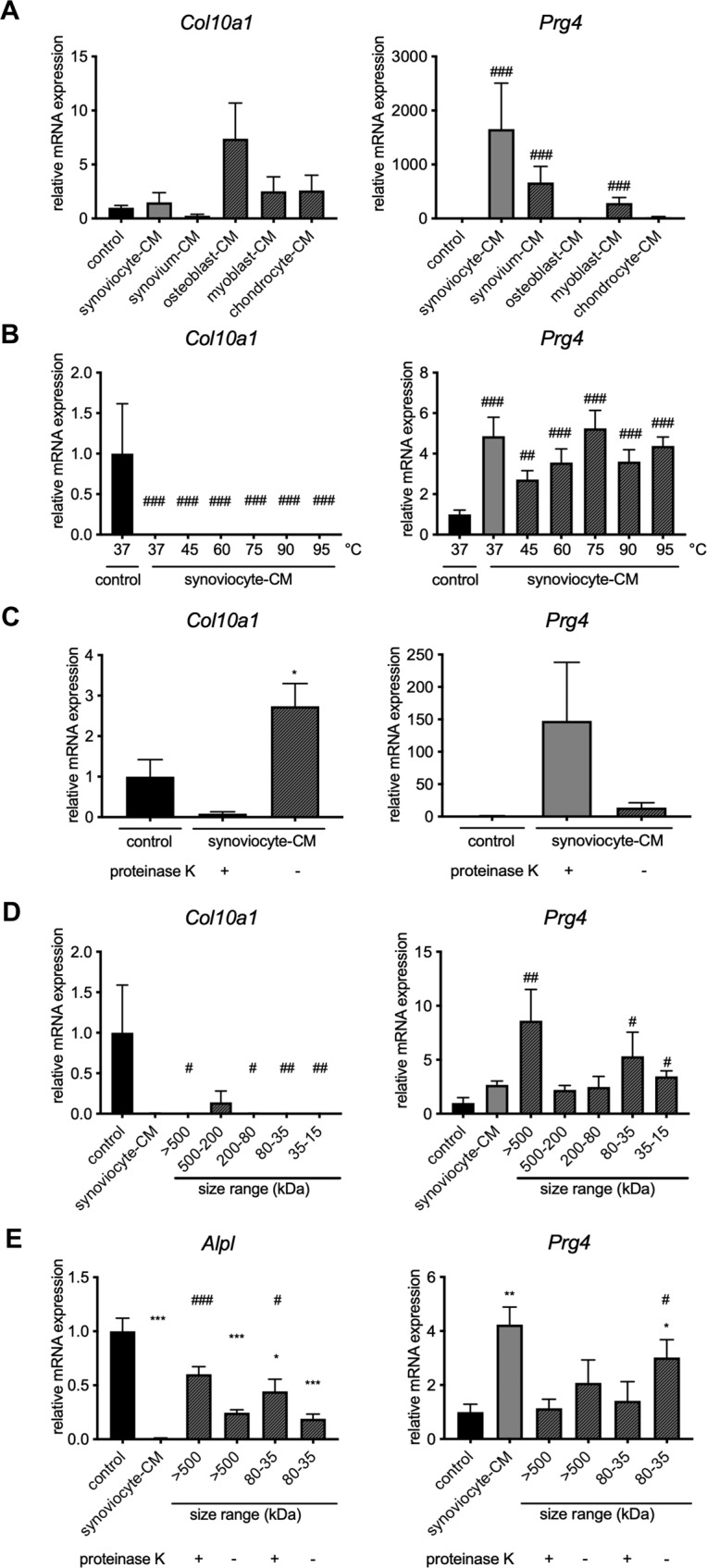
The putative articular cartilage differentiation factor(s) is primarily produced by synoviocytes and is large, temperature-stable protein(s). De-differentiated rat epiphyseal chondrocytes were pelleted and cultured for 7 days in regular chondrogenic media and then either exposed to conditioned media (**A**–**C**) or size-exclusion chromatography fractions (**D**, **E**) of conditioned media (experimental condition) or chondrogenic medium (control) for another 7 days before subjected to gene expression analyses of hypertrophic marker Col10a1 (**A**–**D**) or Alpl (**E**) and articular cartilage superficial zone marker Prg4. **A** Col10a1 and Prg4 expression in chondrocytes at day 14 of culture, in chondrogenic medium (control), or medium conditioned (CM) with rat synovium, rat synoviocytes, rat osteoblasts, rat myoblast, or rat chondrocytes. Expression of Col10a1 and Prg4 in chondrocyte pellets at day 14 of culture in chondrogenic medium (control) or synoviocyte-conditioned medium (synoviocyte-CM) heated to different temperatures (**B**), incubated with proteinase K (**C**), or size-exclusion fractionated (**D**). **E** Alpl and Prg4 expression in chondrocytes at day 14 of culture in chondrogenic media or size-exclusion fractions of synoviocyte-CM digested with proteinase K treated or incubated without proteinase K. Bars represent average ± SEM of 6 biological replicates. **A**–**E** One-way ANOVA followed by Dunnett’s method for relevant pair-wise comparisons.

We next hypothesized that synoviocytes produce a soluble factor or factors that inhibits hypertrophic differentiation and promotes differentiation of articular chondrocytes. To test this hypothesis, we used expanded, primary chondrocytes that were pelleted and cultured (Fig. 8A). Chondrocytes cultured in pellets undergo a reproducible hypertrophic chondrocyte differentiation program^11–13^. As expected, when cultured in chondrogenic media (control), the pellet cultures underwent a chondrocyte differentiation program with increased *Col2a1* and aggrecan expression during the first 7 days (Supplementary figure 3A) followed by hypertrophic differentiation with dramatically increased expression of *Col10a1* (*P* < 0.001, day 21 vs. day 1)*, Alpl*, (*P* < 0.001, day 7, 14, and 21, each compared with day 1) and Indian hedgehog (*Ihh*) (day 14 and 21, both *P* < 0.001 compared to day 1; Fig. 8C*)* and characteristic histological changes including cell enlargement and increased proteoglycan content in extracellular matrix (Fig. 8B) at 14 and 21 days of culture. In contrast, articular cartilage superficial zone marker *Prg4* remained low at all timepoints (Fig. 8C). However, when pieces of rat joint capsule were added to the pellet cultures, hypertrophic differentiation was blocked, and instead *Prg4* expression was dramatically increased (Supplementary figure 3B). Similar results were obtained when pellets were not directly exposed to joint capsule, but instead exposed to media conditioned with pieces of joint capsule (Supplementary figure 3C) demonstrating that effect is due to a soluble factor that does not require cell migration from the synovium or cell-cell contact. In order to refine the model, chondrocyte pellet cultures were then exposed to media conditioned with rabbit synoviocyte cell line HIG82 (from here termed synoviocyte-conditioned media; Fig. 8C). In chondrocyte pellet cultures exposed to synoviocyte-conditioned media, hypertrophic differentiation was inhibited with expression of hypertrophic markers remaining low at day 14 and 21 compared to control media (*Col10a1:* *P* = n.s. and *P* < 0.05 at day 14 and 21, respectively; *AlpI:* *P* < 0.01 and *P* < 0.001 at day 14 and 21, respectively; *Ihh*, *P* < 0.001 and *P* < 0.05 at day 14 and 21, respectively; Fig. 8C), whereas *Prg4* expression was induced (synoviocyte-condition vs. control media, *P* < 0.001 and *P* < 0.05 at day 14 and 21, respectively; Fig. 8C). Interestingly, in situ hybridization experiments indicated that *Prg4* was primarily induced in the most superficial cells of the pellets exposed to synoviocyte-conditioned media (Fig. 8D). Synoviocyte- (*P* < 0.001), synovium- (*P* < 0.01) and myoblast-conditioned (*P* < 0.01) media, but not media conditioned with osteoblasts or chondrocytes induced *Prg4* expression (Fig. 9A), suggesting that synoviocytes are the primary source of this putative synovial articular cartilage differentiation factor or factors.

### The synoviocyte-secreted articular cartilage differentiation factor(s) is a large and temperature-stable protein

In order to characterize basic physical properties of the putative synovial factor, the heat stability of the synovial factor was tested. First, synoviocyte-conditioned media was heated to different temperatures for 10 min. The bioactivity was surprisingly resistant to heating, as conditioned media heated up to 95 °C still were able to significantly suppress *Col10a1* expression (*P* < 0.001, all temperatures vs. control) and induce *Prg4* expression (*P* < 0.01 for 45 °C and *P* < 0.001, for all other temperatures vs. control; Fig. 9B). However, the bioactivity of synoviocyte-CM appeared to be sensitive to proteinase K digestion as proteinase K digested synoviocyte-conditioned media failed to suppress *Col10a1* expression and induce *Prg4* expression compared to undigested synoviocyte-conditioned media (*P* = 0.05 and *P* = n.s., *Col10a1* and *Prg4*, respectively; Fig. 9).

In order to explore the size of the putative factor(s), synoviocyte-conditioned media was subjected to size-exclusion chromatography and the resulting fractions added to chondrocyte pellet cultures. The bioactivity was consistently retained in the 80–35 kDa range, but also in the upper, large fraction (>500 kDa), as shown by the decreased *Col10a1* (*P* < 0.05 and *P* < 0.01 for >500 kDa and 80–35 kDa, respectively; Fig. 9D) and increased *Prg4* expression (*P* < 0.01 and *P* < 0.05 for >500 kDa and 80–35 kDa, respectively; Fig. 9D). The bioactivity detected in each of the two fractions also appeared to be sensitive to proteolytic degradation. After proteinase K digestion, the suppression of hypertrophic chondrocyte marker *Alpl* observed with undigested fractions was attenuated (*P* < 0.001. and *P* = 0.046 for no proteinase *K* vs. proteinase *K* in >500 fraction and 80–35 KDa, respectively; Fig. 9E) with corresponding decreases in *Prg4* expression (*P* = n.s. and *P* = 0.039 for no proteinase K vs. proteinase K in >500 fraction and 80–35 KDa, respectively; Fig. 9E). The finding of bioactivity in two different media fractions could either be due to the existence of 2 or more factors or due to a single factor that exists in both mono- and multimers or protein complexes.

## Discussion

**Fig. 10:**
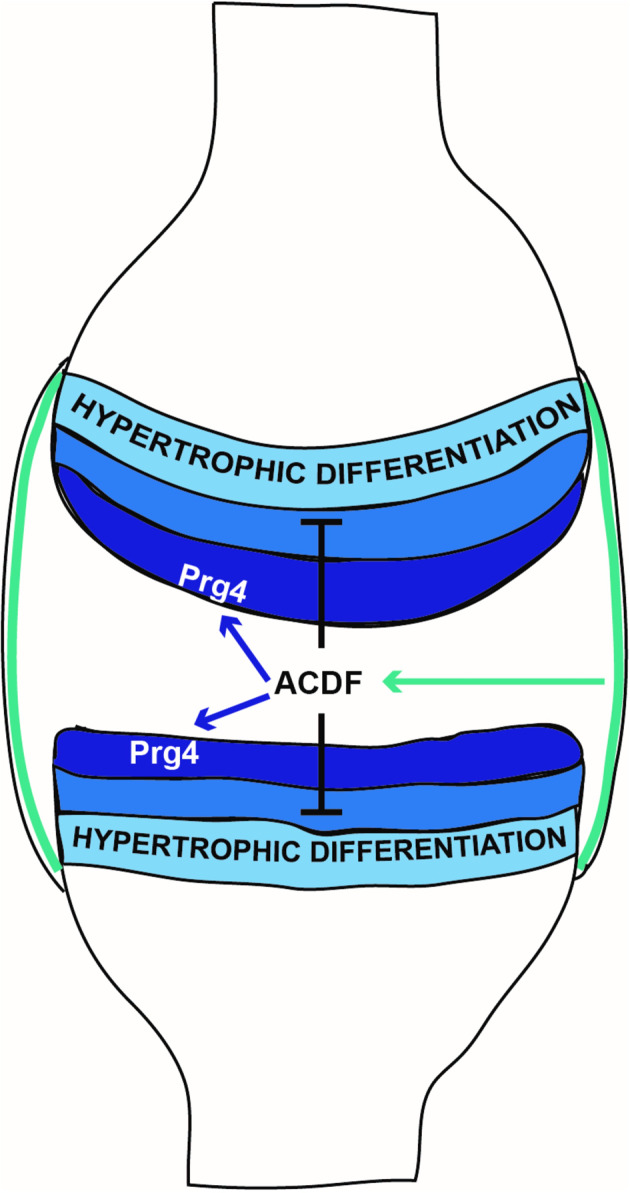
Proposed model: Synoviocytes, via the secretion of articular cartilage differentiation factor (ACDF) affect chondrocyte differentiation at the joint surface. Schematic representation of proposed functional model. Synoviocytes in the synovial membrane (green) secrete articular cartilage differentiation factor(s) (ACDF) to the synovial fluid. ACDF diffuse into the articular cartilage and inhibits hypertrophic differentiation in the superficial (dark blue) and intermediate zone (blue), but do not reach the deep zone (light blue) in sufficient concentration to block hypertrophic differentiation. ACDF also directly or indirectly induce Prg4 (lubricin) expression in the superficial zone. Synoviocyte-produced ACDF may thereby inhibit endochondral bone formation and support articular cartilage formation and maintenance at the joint surface.

The current notion is that articular chondrocytes are inherently different from other chondrocytes of the cartilaginous bone templates, therefore explaining why they are able to escape hypertrophic differentiation and endochondral bone formation. However, an alternative or supplementary explanation might be that synovial fluid contains factors that diffuse into articular cartilage and inhibit hypertrophic differentiation, thereby protecting it from endochondral ossification. In order to explore the role of the synovial microenvironment on chondrocyte differentiation, osteochondral allografts consisting of articular cartilage, epiphyseal bone, and growth plate cartilage from distal femoral epiphyses of rats with ubiquitous eGFP expression were transplanted to matching sites in eGFP-negative littermates. At the joint surface, hypertrophic differentiation was suppressed and growth plate cartilage exposed to the synovial joint microenvironment gradually remodeled into an articular cartilage-like structure, suggesting that the synovial joint microenvironment not only inhibits hypertrophic differentiation but also promotes superficial zone chondrocyte differentiation. This hypothesis was supported and extended by the finding that high-density chondrocyte pellet cultures that normally undergo a robust hypertrophic differentiation program, instead differentiated towards the articular cartilage superficial chondrocyte phenotype when exposed to media conditioned with pieces of rat joint capsule, primary rat synoviocytes, or to a rabbit synoviocyte cell line, but not when exposed to media conditioned with rat osteoblasts or chondrocytes. Taken together, these findings indicate a novel mechanism by which synoviocytes, through the secretion of a factor or several factors, act to inhibit hypertrophic differentiation and promote articular cartilage formation by chondrocytes located at the joint surface (Fig. 10). It may be speculated, that the proposed mechanism, possibly together with mechanical stimuli, support the formation of a superficial zone stem cell niche that enables superficial chondrocytes to become self-renewing unipotent stem cells capable of maintaining and renewing the adult articular cartilage^14^.

Articular cartilage placed ectopically inside the epiphysis and thereby isolated from exposure to the synovial fluid, remained structurally and phenotypically intact at postoperative day 3, but were then gradually remodeled by continuous endochondral bone formation at the epiphyseal side and, interestingly, after 28 days by hypertrophic differentiation in the original superficial zone (now adjacent to the metaphysis). These observations are largely consistent with the hypothesis that synovial fluid acts to stabilize the phenotype of the superficial zone chondrocytes. The importance of synovial fluid to lubricate and protect the superficial zone is well established^15–18^. These studies together with our finding of induced *Prg4* expression in chondrocyte pellet cultures exposed to synoviocyte-conditioned media suggest that epiphyseal chondrocytes exposed to synovial fluid are directed towards the superficial zone phenotype at least in part by factors in the microenvironment. This would make evolutionary sense as it would allow underlying intermediate zone chondrocytes to replace superficial zone cells in case of injury to the superficial zone and could help explain why superficial articular cartilage injuries tend to heal well despite the loss of superficial chondrocytes. Similarly, it may provide an explanation for the finding that ablation of *Prg4*-positive superficial zone chondrocytes only had modest effects on articular cartilage function unless killing was extensive^19^.

The proposed mechanism (Fig. 10) may also have important physiological roles during joint formation and could help explain why the only chondrocytes of the cartilaginous bone templates that are spared from endochondral bone formation during skeletal development are those exposed to synovial fluid. It might also contribute, together with mechanical motion and shear stress^20^ to the observation that lubricin (*Prg4*) expression by interzone cells is not induced until joint cavitation has occurred^21,22^. The findings also provide support for the hypothesis that impairment of the synovial microenvironment is an important pathogenic mechanism for osteoarthritis and other degenerative joint disorders^23–26^.

We have not yet identified the putative factor/factors and potential candidates are numerous as chondrocyte differentiation is regulated by a complex network of paracrine signals, including Wnts, Bone morphogenetic protein (Bmp)/Transforming growth factor-β (Tgfβ), Parathyroid hormone-related peptide (PTHrP), Indian hedgehog (IHH), Fibroblast growth factors (Fgf), Insulin like growth factor (Igfs) and Hypoxia-inducible factor (Hif) 1^,^^27^. For example, Wnt5a and Wnt5b are promising candidates as they are both able to stimulate *Prg4* expression in superficial zone chondrocytes^28^. The molecular sizes of rabbit Wnt5a and Wnt5b are 42,3 kDa and 49,6 kDa, respectively, and are thus both within the 80–35 KDa size range. Other examples of potential candidates are BMP-antagonists. Hypertrophic differentiation is promoted by BMPs and inhibited by the BMP receptor antagonist Noggin^29^. Noggin has a molecular size of 46 kDa and may thus be a candidate, at least as an inhibitor of hypertrophic differentiation. However, it appears to be more abundantly expressed in epiphyseal cartilage than in the synovial lining of the joint capsule, at least in fetal mice limbs^30^. Chordin (120 kDa) is a BMP antagonist that is too large to be a potential candidate for the 35 – 80 kDa fraction but might be a potential candidate for the >500 kDa fraction if it exists as a multimer (tetramer or larger) in synoviocyte-conditioned media. Sclerostin (22 kDa) and Gremlin (28 kDa) cannot be completely excluded but are perhaps less likely given their smaller sizes. Similarly, PTHrP, an important physiological inhibitor of hypertrophic differentiation exists in 20 kDa and 24 kDa isoforms, which makes it less likely as a candidate. Also, its pattern of expression does not fit the expected pattern of expression for the putative factor as it is expressed in articular cartilage and do not appear to be expressed in the synovial membrane^31^.

The study has several limitations, including those inherent to in vivo animal models and in vitro experiments. Firstly, although rodent growth plate cartilage considerably slows its growth rate, it does not completely fuse until the later part of the lifespan of the animal. It is unknown whether this physiological difference in rat compared to human growth plate offers a greater capacity for cell maintenance and/or differentiation. Secondly, our study presents phenotypic and transcriptional changes in the transplanted cartilage, but it does not assess certain functional changes, specifically the secretion of lubricin protein or changes in biomechanical properties. However, BrdU labeling and staining was able to show areas of active cell proliferation at the articular surface of inverted allografts and TUNEL staining did not indicate increased apoptosis in the transplants compared to the surrounding tissue of the recipient. Thirdly, while an in vivo animal model has the benefit of simulating the natural synovial microenvironment it is unable to isolate independent factors, such as biochemical versus biomechanical factors, which limits it to a proof-of-concept and hypothesis generating experiment. The addition of in vitro experiments enabled us to show evidence of a biochemical factor. However, it did not allow us to also recapitulate biomechanical factors, which was beyond the scope of this study. Future directions are to identify the putative synoviocyte factor and confirm its effect on chondrocyte differentiation and explore its potential role in development, maintenance and regeneration of articular cartilage.

In summary, we transplanted osteochondral allografts containing articular cartilage at one end and growth plate cartilage at the other end in original or inverted orientation into distal femoral epiphyses of 4-week-old rats. Our model allowed the tracing and characterization of transplanted cells. We found that growth plate cartilage transplanted to the articular surface remodeled into articular-like cartilage and that hypertrophic differentiation was inhibited at the articular surface. These observations were confirmed using a rat chondrocyte pellet culture model. Our results taken together suggest that a factor or several factors in the synovial joint microenvironment are able to inhibit hypertrophic differentiation and promote differentiation towards the superficial articular chondrocyte phenotype. This novel mechanism and the future identification of the putative factor(s) may have important clinical implications for understanding articular cartilage development, the pathophysiology of degenerative articular cartilage diseases, as well as for biomedical engineering of articular cartilage.

## Methods

### Animals

All animal procedures were approved by the Animal Care and Use Committee, National Institute of Child Health and Human Development (Animal Study Proposal number: 13-087) and the regional animal ethics committee in Stockholm (Permit no. N248/15, N118/16, and 15635-2017). Animals were housed under standard conditions in a 12-hour light/dark cycle and provided standard rat chow and water ad libitum. Animal care of this study complied with EU directive 2010/63/EU for the protection of animals used for scientific purposes. In order to trace transplanted cells, inbred Lewis rats overexpressing enhanced green fluorescent protein (eGFP) under the ubiquitous CAG promoter (Lew-Tg(CAG/eGFP)YsRrrc; Rat Research and Resource Center, Columbia, MO, USA) were bred to wildtype resulting in a 1:1 ratio of transgenic hemizygous to wildtype animals. Four-week-old *eGFP*-positive animals were used as donors and wildtype littermates of the same age as recipients. Before starting the surgery experiments, two animals were used for protocol optimization and were not included in the analyses. During 18 surgical sessions, equal number of animals were randomly distributed into the inverted transplantation group (with GP Flip) and the original transplantation group (with AC transplant rather than GP Flip) over the three timepoints (day 3, 7, and 28) regardless of animal gender. Additionally, we expected obvious phenotypic difference between the experimental and the control group. The number of animals were therefore limited to three per timepoint in each group considering the 3 R principles. For chondrocyte isolation, epiphyseal cartilage was dissected from distal femurs and proximal tibias of 3–5-day-old Sprague Dawley rats (Charles River Laboratory, Kuopio, Finland). Animals were housed under standard conditions with a 12-hour light/dark cycle and received standard rat chow and freshwater ad libitum.

### Osteochondral allograft transplantation

Four-week-old wildtype and eGFP-transgenic rats were premedicated with atropine (0.02–0.05 mg/kg), ketamine (75 mg/kg), and acepromazine (2.5 mg/kg) or alternatively directly sedated with isoflurane. Knees were clipped and scrubbed with 70% isopropyl alcohol, petrolatum eye ointment was administered to each eye, and normal saline (5–10 ml SQ), ketofen (3–5 mg/kg), or buprenorphine (20 mcg/kg) and infection prophylaxis with cefazolin (25 mg/kg) were administered. The animals were placed under general anesthesia using mask isoflurane. Knee joints were opened by a vertical incision lateral to the patella and the distal femoral condyles were exposed. Using 17-guage PercuCut bone biopsy needles (Bracco Diagnostics, Inc., Princeton, NJ, USA) an osteochondral graft consisting of articular cartilage, epiphyseal bone, growth plate cartilage, and metaphyseal bone was extracted from the distal femoral intercondylar groove. For donor grafts, metaphyseal bone was sharply removed by a transverse cut through the hypertrophic zone under a dissecting microscope and the removed tissue piece was collected for histological analysis. After donor grafts were collected, donor animals were euthanized by an intracardial injection of 0.5 ml of euthanasia solution (MWI Veterinary Supply, Boise, ID) containing pentobarbital sodium (3.9 mg/ml) and phenytoin sodium (0.5 mg/ml). The trimmed eGFP-positive grafts were transplanted into the distal femoral epiphysis of eGFP-negative recipients, either inverted to place growth plate cartilage at the joint surface, or original orientation placing articular cartilage at the joint surface. The joint capsule and skin were closed in layers using 7.0 vicryl sutures. Marcain® (2.5 mg/ml; 0.2 ml SQ) was injected around the surgical scar for local anesthesia. After the completion of surgery, buprenorphine (10–20 mcg/kg) were administered every 12 h for the first 48 h. At post surgery day 3, 7, and 28, animals received an injection of BrdU (50 mg/kg) 4 h before being euthanized by carbon dioxide inhalation. Distal femoral epiphyses were rapidly excised, fixed (10% formalin) overnight, photographed with a Nikon SMZ 1500 dissecting microscope (Nikon Instruments, Inc., Melville, NY), decalcified (15% EDTA, 0.5% PFA) for 3–6 weeks, embedded in paraffin, and sectioned (6 μm thick) for histological analysis, immunohistochemistry, and in situ hybridization.

### Tracing of transplanted cells using immunohistochemistry

Immunohistochemistry was conducted as previously described^32,33^. Briefly, paraffin-embedded tissue sections were baked at 65 °C for 1 h, deparaffinized in xylene, rehydrated through an ethanol series (100%, 100%, 95%, and 70%), and rinsed in PBS. Antigen retrieval was performed with proteinase K (10 μg/ml in PBS) at room temperature for 15 min. Endogenous peroxidase activity was blocked by immersing tissue sections in 3% H_2_O_2_ at room temperature for 15 min. Primary antibodies (anti-GFP antibody ab290, anti-BrdU antibody ab152095, Abcam, Cambridge) were incubated at 4 °C overnight. Staining was performed using VECTASTAIN® ABC Kit (Vector Laboratories, Burlingame, CA) followed by a DAB Substrate Kit (Vector Laboratories, Burlingame, CA) according to the manufacturer's instructions. All wash steps were carried out using TBS with 0.1% Tween. Tissue sections were counterstained with methyl green, fast red, hematoxylin-eosin (Vector Laboratories, Burlingame, CA), dehydrated in an ethanol series (95%, 100%, and 100%), cleared in xylene, and mounted in permount (Fisher Scientific, Fair Lawn, NJ).

### In situ hybridization of chondrocyte differentiation markers

Once the transplanted osteochondral graft was localized by GFP immunohistochemistry, in situ hybridization for chondrocyte markers *Prg4*, *Col10a1*, *Col2a1*, and *Col1a1* was performed on consecutive sections. The gene sequences for rat *Col1a1* (bone and chondrocyte de-differentiation marker)*, Col2a1* (chondrocyte marker) *Col10a1* (hypertrophic chondrocyte marker), and *Prg4* (superficial chondrocyte marker) were obtained from the UCSC Genome Browser. Primers were designed using Primer-Blast (https://www.ncbi.nlm.nih.gov/tools/primer-blast/), and the resulting amplicons were confirmed by NCBI Nucleotide Blast. DNA templates for riboprobe transcription were amplified by PCR using Platinum Taq DNA Polymerase (Invitrogen), cDNA reverse transcribed from total RNA isolated from 3-day-old rat proximal tibial epiphyses as previously described^34^, and primers according to Supplementary Table 1 with forward primers containing a T7 promoter (5′-TAATACGACTCACTATAGGGAG-3′), and reverse primers containing an Sp6 promoter (5′-TGGATTTAGGTGACACTATAGAAG-3′).

PCR of DNA templates was performed with a 2720 Thermal Cycler (Applied Biosystems, Waltham, MA) using the following parameters: hold at 94 °C for 5 min, followed by 30 cycles of denaturing at 94 °C for 30 sec, annealing at 58 °C for 30 sec, and extending at 72 °C for 45 sec, followed by a final extension at 72 °C for 3 min. PCR products were purified by agarose gel electrophoresis and a QIAquick Gel Extraction Kit (Qiagen, Hilden, Germany). A second PCR was performed using the same parameters and the products were purified with a QIAquick PCR Purification Kit (Qiagen). Single-stranded riboprobes were transcribed with a DIG Labeling Kit (Roche, Basel, Switzerland) incorporating a digoxigenin (DIG)-conjugated uracil every 20–25 nucleotides. Sp6 polymerase was used for antisense strand riboprobes and T7 polymerase was used for sense strand riboprobes. Riboprobes were purified with Micro Bio-Spin 30 Columns (Bio-Rad) and quantified with a NanoDrop Spectrophotometer (Thermo Fisher Scientific, Waltham, MA).

After the preparation of DIG-labeled riboprobes, non-radioactive digoxigenin in situ hybridization was performed as previously described with slight modifications^33,35^. The detailed protocol is available upon request. Briefly, tissue sections were baked at 65 °C for 1 hr, deparaffinized in xylene, rehydrated through an ethanol series (100%, 100%, 95%, and 70%), and rinsed in DEPC-treated water. Tissue sections (6 μm) were permeabilized with proteinase K at room temperature for 15 min (10 μg/ml in PBS, pH7.4), postfixed for 5 min (10% formalin), and acetylated for 15 min (0.25% acetic anhydride in 0.1 M triethanolamine) with each step followed by two 5 min washes in PBS. Prehybridization was carried out at 65 °C for 2 h in hybridization solution (50% formamide, 10 mM Tris pH7.6, 200 μg/ml Torula yeast RNA, 1× Denhardt’s solution, 10% dextran sulfate, 600 mM NaCl, 0.25% SDS, 1 mM EDTA, pH8.0). Hybridization with DIG-labeled riboprobes (100 ng in 100 μl hybridization solution) was performed at 65 °C for 4 h. Posthybridization was carried out by washing with 50% formamide in 1× SSC at 65 °C for 30 min, digesting with RNase A (20 or 2000 μg/ml in 1 M NaCl, 10 mM Tris HCl, 1 mM EDTA, pH8) at 37 °C for 30 min, and washing in SSC at increasing stringency (4×, 1×, 0.5×, and 0.2×). For detection of hybridized riboprobes, tissue sections were rinsed in MABT (0.1 M maleic acid, 0.15 M NaCl, 0.1% v/v Tween-20, pH7.5), blocked with 1% BSA in MABT at room temperature for 30 min, incubated with alkaline phosphatase-conjugated anti-DIG antibody (Roche-11093274910) in 1% BSA in MABT at 4 °C for overnight, and, on the next day, incubated with nitro blue tetrazolium chloride/5-bromo-4-chloro-3-indolyl phosphate (NBT/BCIP) substrates (Sigma-Aldrich) in NTM (100 mM NaCl, 100 mM Tris pH9.5, 50 mM MgCl_2_) at room temperature protected from light until a colorimetric change was detected. To mount, tissue sections were rinsed in PBS for 5 min, fixed in 10% formalin for 20 min, counterstained with nuclear fast red, dehydrated in an ethanol series (70%, 95%, and 100%), cleared in xylene, and mounted with Permount.

Staining was visualized by scanning the slides under bright-field microscopy with a Panoramic MIDI II 2.0.5 digital scanner (3DHISTECH Ltd, Budapest, Hungary).

### Histomorphometry

High-resolution images of 6 μm sections were obtained using either Panoramic MIDI II 2.0.5. scanner or ScanScope CS digital scanner (Aperio Technologies, United States). After calibration, measurements were obtained using Caseviewer (3DHISTECH) and ImageScope (Aperio) softwares. The analysis was carried out in the recipient femoral epiphyses (*n* = 3 for each timepoint) of the recipient growth plate (native GP), recipient articular cartilage (native AC), growth plate cartilage ectopically placed at the joint surface (GP flip), and articular cartilage ectopically placed inside the epiphysis (AC flip) on Masson’s trichrome stained slides (*n* = 3, per group and timepoint). All measurements were performed on one slide per sample. For thickness/height measurements of different tissues, 10 individual measurements evenly distributed over the transplanted tissues were obtained and averaged unless stated otherwise.

In order to quantitate the remodeling resulting from hypertrophic inhibitory effect of a putative synovial factor, the distance from the articular surface to the first *Col10a1*-expressing cell in native AC and GP flip and the distance from the edge of the secondary ossification center to 30 representative Col10a1-expressing cells in native AC and GP flip were assessed (*n* = 3 per timepoint).

### Cell apoptosis assay

Apoptotic cells were detected on paraffin sections using the TUNEL (terminal deoxynucleotide transferase-mediated dUTP nick end labeling) assay (FragELTM DNA Fragmentation Detection Kit, EMD Millipore), according to the manufacturer’s protocol. Briefly, sections were subjected to Proteinase K treatment (20 µg/mL proteinase K in Tris pH8, 20 mins RT) and followed by incubation with Fluorescein-FragELTM TdT Labeling Reaction Mix (90 mins, 37 °C). Post incubation, sections were rinsed, and mounted with Fluorescein-FragELTM mounting media, containing 4,6-diamidino-2-phenylindole counterstain. For each sample (one section per sample), TUNEL positive and negative cells in six randomly selected areas located in the biopsy, close to the biopsy, and far from the biopsy were counted and the percent of positive cells was calculated after adding the counts from each of the six areas.

### Isolation and culture of chondrocyte pellets

Epiphyseal chondrocytes were isolated from proximal tibial and distal femoral epiphyses of 3–5-day-old rats (Sprague Dawley, Charles River Laboratory, Kuopio, Finland), digested with 0.3% collagenase type IA (Sigma-Aldrich) in DMEM/F12 in sterile conditions as previously described^13^. The resuspended chondrocytes were then seeded at 20 × 10^3^ cells/cm^2^ and expanded in DMEM/F12 with GlutaMAX (Thermo Fisher Scientific, Waltham, MA, USA) with 10% fetal bovine serum (FBS), 1% penicillin/streptomycin, 50 ng/ml fungizone, and 50 μg/ml ascorbic acid (all by Thermo Fisher Scientific, Waltham, MA, USA), referred to as chondrogenic medium, at 37 °C and 5% CO_2_. Confluent cultures were trypsinized and chondrocytes were frozen and stored at −196 °C until use. To make pellet cultures, chondrocytes were thawed and plated at approximately 15 × 10^3^ cells/cm^2^. Confluent chondrocytes were washed, trypsinized, and pellet cultures were formed by spinning 2.0 × 10^5^ viable chondrocytes in round bottom ultra-low attachment 96-well microplates (Corning, Sigma-Aldrich, Darmstadt, Germany) at 500 × *g* for 5 min at room temperature. Chondrogenic medium was refreshed every other day. From day 7 of culture onwards, chondrocyte pellets were cultured in conditioned media (100%), (details are described in the following paragraph), or control (chondrogenic medium: DMEM/F12 with GlutaMAX (Thermo Fisher Scientific, Waltham, MA, USA) with 10% FBS, 1% penicillin/streptomycin, 50 ng/ml fungizone, and 50 μg/ml ascorbic acid (all by Thermo Fisher Scientific, Waltham, MA, USA)) and collected at day 1, 7, 14, and 21 for RNA extraction and real-time PCR, or fixed (4% PFA at 4 °C) for histological analyses and in situ hybridization. For experiments of chondrocyte pellets cultured with synovium tissue (Supplementary figure 3B), epiphyseal chondrocytes were isolated from 10-day-old rats following the same procedure as previously described, pelleted and cultured in chondrogenic media (control) for 14 days, or with synovium tissue from adult rats from day 7 to day 14 of culture. RNA was extracted on day 3, 7, and 14. For experiments of chondrocyte pellets cultured with medium conditioned with synovium tissue (Supplementary figure 3C), pellets were cultured in chondrogenic media (control) for 14 days or with medium conditioned with synovium (50% and 100%) from day 7 to 14, and pellets collected for gene expression analysis at day 1, 3, 7, and 14.

### Conditioned media

Rabbit fibroblast-like synoviocyte cell line HIG82 (ATCC® CRL1832™)^36–38^ was also used to produce synoviocyte-conditioned medium by culturing HIG82 in Ham’s F-10 Nutrient Mixture (Sigma-Aldrich, Darmstadt, Germany, lot number: 1830067) with 10% FBS, 1% penicillin/streptomycin, and 50 ng/ml fungizone (all by Thermo Fisher Scientific, Waltham, MA, USA). When confluence was reached, the cells were washed once and incubated with the above media except that FBS depleted of large proteins using a 50 kDa spin filter (Spin-X UF 20 Concentrator, 50 kDa MWCO, Corning, Sigma-Aldrich, Darmstadt, Germany) was used. Conditioned media were collected every other day for 6 weeks, by spinning at 1000 × *g* for 5 min, followed by 3000×*g* for 10 min. Pooled conditioned media were concentrated 25-times using spin filters (Spin-X UF Concentrator, 5 kDa MWCO, Corning, Sigma-Aldrich, Darmstadt, Germany) and diluted 1:10 in chondrogenic medium (DMEM/F12 with GlutaMAX with 10% FBS, 1% penicillin/streptomycin, 50 ng/ml fungizone, and 50 μg/ml ascorbic acid (all by Thermo Fisher Scientific, Waltham, MA, USA)) before addition to chondrocyte pellet cultures. From here on ‘synoviocyte-conditioned media’ refers to media conditioned with HIG82 cells.

Conditioned media were produced also by synoviocytes, synovium, osteoblasts, myoblasts, and epiphyseal chondrocytes isolated from 20-day-old rats (Sprague Dawley, Charles River Laboratory, Wilmington, MA, USA), and cultured in monolayer to produce conditioned media. In detail, synoviocytes were isolated from synovial tissues after mincing and digestion with collagenase 1 A (1 mg/ml, Sigma-Aldrich) for 2 h at 37 °C. The resuspended synoviocytes were then seeded and expanded as monolayer in DMEM/F12 with GlutaMAX (Thermo Fisher Scientific, Waltham, MA, USA) with 10% FBS, 1% penicillin/streptomycin, 50 ng/ml fungizone, (all by Thermo Fisher Scientific, Waltham, MA, USA). Myoblasts were isolated from quadriceps, dissociated with 0.25% trypsin (Thermo Fisher Scientific, Waltham, MA, USA) for 20 min at 37 °C, and cultured in the media mentioned above. Chondrocytes were isolated and expanded as previously described. Osteoblasts were isolated from femurs and tibia after removing connective tissues and periosteum. After washing with PBS, bone pieces were transferred into culture in DMEM/F12 with GlutaMAX (Thermo Fisher Scientific, Waltham, MA, USA) with 10% FBS, 1% penicillin/streptomycin, 50 ng/ml fungizone, and 50 μg/ml ascorbic acid, (all by Thermo Fisher Scientific, Waltham, MA, USA) to allow cells to migrate out. Bone pieces were removed at first medium change, and cells cultured until confluent. The same culture conditions were adopted for synovium and the nature of all cell type was visually inspected. When confluence was reached, all cells except myoblasts were cultured with FBS depleted of large proteins as previously described, and conditioned medium collected every other day for four weeks, stored at −20 °C, pooled and sterile filtered, before addition to chondrocyte pellet cultures.

In order to explore the heat stability of the synovial factor, synoviocyte-conditioned media underwent heat treatment at 45 °C, 60 °C, 75 °C, 90 °C, or 95 °C for 10 min followed by 3 min at room temperature and centrifugation at 21,000 × *g* for 60 min. The supernatants were then concentrated and diluted as described above before addition to chondrocyte pellet cultures.

To determine the size of the synovial factor, synoviocyte-conditioned medium was separated into fractions by using Size-Exclusion Chromatography (Äkta Pure FPLC, GE Healthcare technology) with a Superdex 200 Increase 10/300 GL column (GE Healthcare technology). Fractions were then concentrated to 200 μl and diluted 1:10 in chondrogenic medium (DMEM/F12 with GlutaMAX with 10% FBS, 1% penicillin/streptomycin, 50 ng/ml fungizone, and 50 μg/ml ascorbic acid (all by Thermo Fisher Scientific, Waltham, MA, USA)) before addition to chondrocyte pellet cultures. In order to assess the specific size range of each fraction, a calibration kit (Gel filtration calibration kit, GE healthcare technology) was run in parallel and size was calculated according to the manufacturer’s instructions.

In order to determine whether the putative articular cartilage differentiation factor is a protein, synoviocyte-conditioned media-fractions (>500 KDa and 80–35 KDa) were treated with 0.2 mg/ml proteinase K (Thermo Fisher Scientific, Waltham, MA, USA) for 20 min at room temperature followed by heat inactivation of proteinase K at 75 °C for 10 min, then concentrated, diluted and added to chondrocyte cultures.

In situ hybridization of *Col10a1* and *Prg4* was performed as detailed above on sections of paraffin-embedded pellets collected after 14 days of culture.

### Quantitative real-time PCR of chondrocyte marker expression

Chondrocyte pellets were collected in solution C (4 M guanidine thiocyanate, 25 mM sodium citrate pH7, 0.1 M β-mercaptoethanol) at day 1, 7, 14, 21 of culture and total RNA extracted using phenol chloroform, as previously described^13^. Total RNA (50 ng) was reverse-transcribed into cDNA by Superscript Reverse Transcriptase IV (Thermo Fisher Scientific, Waltham, MA, USA). Real-time PCR (ABI Prism 7900 Fast Sequence Detector, Thermo Fisher Scientific, Waltham, MA, USA) was used to quantify chondrocyte markers Prg4, Alpl, Ihh, Col10a1, Col2a1, Acan (Supplementary Table 2). Data were normalized to 18 S rRNA (eukaryotic 18 S rRNA endogenous control, Thermo Fisher Scientific, Waltham, MA, USA) and relative expression was calculated as previously described using the formula: 2 −ΔCt × 106, with ΔCt being target gene expression relative to 18 S rRNA. The endogenous control was validated versus Gapdh and found to be similar to Gapdh at the conditions used in the study (Supplementary Figure 3D).

### Statistical analysis

All data are expressed as mean ± SEM. For quantitative histology, the overall effect of time and tissue was assessed by two-way ANOVA followed by pair-wise comparisons using Tukey’s multiple comparisons test. Chondrocyte pellet culture were repeated at least three times. For statistical analyses, data from two representative experiments (*n* = 6 biological replicates) were pooled. Real-time PCR relative expression data were log-transformed and the effect of time, the effect of conditioned media as well as the effect of temperature each evaluated using one-way ANOVA and relevant pair-wise comparisons assessed by Student’s *t* tests corrected for multiple comparisons using Dunnett’s method. All statistical calculations were performed using GraphPad Prism 8.0 (GraphPad Software, Inc., La Jolla, CA, USA). All *P* values were two-tailed, and significance was recognized at *P* < 0.05. To avoid cluttering, *P* > 0.05 were not included in figures unless stated.

### Reporting summary

Further information on research design is available in the Nature Research Reporting Summary linked to this article.

## Supplementary information

Supplementary Tables & Figures

REPORTING SUMMARY

## Data Availability

All data included in this study are available from the corresponding author upon request.
