# Supplementary material for: The synovial microenvironment suppresses chondrocyte hypertrophy and promotes articular chondrocyte differentiation
Source: NPJ Regen Med. 2022 Sep 16;7:51. doi: 10.1038/s41536-022-00247-2 (PMC9481641; doi:10.1038/s41536-022-00247-2)
Supplement: Supplementary file 1 — Supplementary Tables & Figures [file 41536_2022_247_MOESM1_ESM.pdf]

Supplementary Tables and Figures

The synovial microenvironment suppresses chondrocyte hypertrophy and promotes articular chondrocyte differentiation

Michael Chau*, Zelong Dou*, Marta Baroncelli, Ellie B. Landman, Ameya Bendre, Masaru Kanekiyo, Alexandra Gkourogianni, Kevin Barnes, Lars Ottosson, and Ola Nilsson.

* M.C. and Z.D. contributed equally.

Supplementary Table 1. List of primers used to prepare riboprobes for *in situ* hybridization.

Target gene	Primer sequence
Rat ***Col1a1*** cDNA (2618-2968 of GenBank accession no. BC133728)	
Forward primer	5’-CATTGGTAACGTTGGTGCTCCT-3’
Reverse primer	5’-TCTCCTCTCTGACCGGGAAGA-3’
Rat ***Col2a1*** cDNA (3678-4028 of GenBank accession no. L48440.1)	
Forward primer	5’-CTTGAGACAGCATGACGTCGA-3’
Reverse primer	5’-CCGTCGCCGTAGCTGAAGT-3’
Rat ***Col10a1*** cDNA (3601-4002 of GenBank accession no. XM_001053056)	
Forward primer	5’-AAGAGATTTCAGTAAGAGGAGAACAAGG-3’
Reverse primer	5’-TCTGTCCATTCACACCAGGAG-3’
Rat ***Prg4*** cDNA (1593-1943 of GenBank accession no. NM_001105962)	
Forward primer	5’-CCCCTAAGGAGCCCACATCTAC-3’
Reverse primer	5’-GAGTGGTGGTACTTGCTCTTGGTT-3’
Rabbit ***Prg4*** cDNA (1002-1752 of GenBank accession no. XM_002717684.1)	
Forward primer	5’-GCCTCTGACACCTGCTCCTACT-3’
Reverse primer	5’-CTCCTCTGCACCTTCGTTCTTT-3’
Rat ***Bglap*** (79-429 of GenBank accession no. M11777.1)	
Forward primer	5’-TCTCTGACCTGGCAGGTGC-3’
Reverse primer	5’-CCGTCCATACTTTCGAGGCA-3’
Rat ***Dmp1*** (64-414, 146-496, 788-1138 of GenBank accession no. BC129082)	
Forward primer 1	5’-CTGTCGCCAGATACCAAAATACTG-3’
Reverse primer 1	5’-CTTGAGACGTGCTGTCTTCACTG-3’
Forward primer 2	5’-GGCAAATAGTGACCACACGGA-3’
Reverse primer 2	5’-GAGTCTCCTGCCTCAGGCC-3’
Forward primer 3	5’-CGAGCTCGCTGACAGCAAC-3’
Reverse primer 3	5’-TCGCTGTCACCTTGCTCCT-3’

Supplementary Table 2. List of primers for qPCR.

Target gene	Primer sequence
Rat ***Prg4*** (SYBR green)	
Forward primer	5'-GCATTAACATCCATCCCATGTTT-3'
Reverse primer	5'-CCATCCACTGGCTTACCATTG-3'
Rat ***Alpl*** (SYBR green)	
Forward primer	5'-CCAACTCATTTGTGCCAGAGAA-3'
Reverse primer	5'-GAGTTTTTGGAGTTTCAGGGCA-3'
Rat ***Ihh*** (SYBR green)	
Forward primer	5'-TCAGACCGCGACCGAAATAA-3'
Reverse primer	5'-CCGAGTGCTCAGACTTGACAGA-3'
Rat ***Col10a1*** (Taqman, Thermo Fisher Scientific, Waltham, MA, USA)	Rn01408030_m1
Rat **Col2a1** (SYBR green)	
Forward primer	5'-GCCAGGATGCCCGAAAATTAG-3'
Reverse primer	5'-CCACCAGCCTTCTCGTCAAA-3'
Rat **Acan** (SYBR green)	
Forward	5'-GTGCGCCCATCATCAGAAAC-3'
Reverse	5'-GGTGCTTGGACAGTGGATCA-3'
Rat **Gapdh** (SYBR green)	
Forward	5'-TGGTGAAGGTCGGTGTGAAC-3'
Reverse	5'-GGGATCTCGCTCCTGGAAGATG-3'
18S rRNA endogenous control, (Taqman, Thermo Fisher Scientific, Waltham, MA, USA)	X03205.1

Supplementary Table 3. List of critical reagents and tools used during animal knee surgery.

**Product name**	**Source and Catalog**	**Application**	**Dosage**	**Application specific details**
Attane Vet (Isoflurane)	*Piramal Healthcare UK Limited, Northumberland, UK*	*Animal anaesthesia*	*4.5% for initial anaesthesia and 1.5% for mask Anaesthesia*	*Start with 4.5%. Decreased to 1.5% just before transfer the animal from box (O2: 1.0, Air: 1.0) to surgery table under mask (O2: 0.25, Air: 0.25). The ratio 2.5: 2.0 works well for rats.*
Occulentum simplex (Petrolatum eye ointment)	*APL, Stockholm, Sweden*	*For eye lubrication and protection during animal knee surgery*	*NA*	*As instructed*
Normal saline	*B. Braun Melsungen AG, Melsungen Germany*	*To replace fluids loss during animal knee surgery*	*Calculation: Body weight (grams) x % dehydration (generally 10%) = Fluid volume (ml) e.g. 300 gram rat / estimated 10% dehydrated: 300 g X 0.10 = 30 ml of fluids to be replaced.*	*Subcutaneous injection which can be divided to two times for volume larger than 10mL*
17-guage PercuCut 559 bone biopsy needles	*Bracco Diagnostics, Inc., Princeton, NJ, USA*	*For biopsy cylinder harvest and implantation*	*NA*	*Sterilization before use*
7.0 vicryl sutures	*Ethicon, Somerville, NJ, USA*	*For joint capsule and skin closure*	*NA*	*As instructed*
Marcain	*Aspen Pharma Trading Limited, Dublin, Ireland*	*Applied around the surgical scar for local anesthesia post-surgery*	*2.5 mg/ml*	*Subcutaneous injection*
Temgesic (Buprenorphine)	*Indivior Europe Limited,Dublin, Ireland*	*Pain killer applied post-surgery*	*20 mcg/kg*	*Administered every 12 hrs for the first 48 hrs*

Supplementary Table 4. List of reagents used for histological staining, immunohistochemistry (IHC), and *in situ* hybridization (ISH).

**Epitope/Antigen or Product name**	**Source and Catalog no.**	**Host species**	**Application**	**Dilution**	**Application specific details**
Trichrome Stain (Masson) Kit	*Sigma, HT15-1KT*	*NA*	*Masson's trichrome staining*	*NA*	*Per instructions*
Safranin O	*Sigma, 1.15948*	*NA*	*Safranin O mapping*	*Safranin O powder 2,5g, distillated water 250ml*	*Incubation for 30 min*
Fast green	*Sigma, 1.04022*	*NA*	*Counter stain of Safranin O*	*0,05g of Fast Green powder +250ml of distillated water*	*Incubation for 5-8 min*
Anti-GFP primary antibody	*Abcam, ab290*	*Rabbit*	*IHC*	*1:1000*	*Antigen retrieval with 10ug/mL Proteinase K in PBS, followed by blocking in 10% Goat serum in 0.1% TBST, 1hr, RT. Incubation at 4 °C overnight*
Anti-BrdU primary antibody	*Abcam, ab152095*	*Rabbit*	*IHC*	*1:500*	*Antigen retrieval with 10ug/mL Proteinase K in PBS, followed by blocking in 10% Goat serum in 0.1% TBST, 1hr, RT. Incubation at 4 °C overnight*

Biotinylated goat anti-rabbit IgG secondary antibody	*Vector Labs PK-4001*	*Goat*	*IHC*	*1:200*	*Incubation in 0.1% TBST, 1hr RT*
Anti-Digoxigenin-AP, Fab fragments	*Roche 11093274910*	*Sheep*	*ISH*	*1: 2500*	*Blocking in buffered BSA 1% at RT for 30min, and incubation at 4 °C overnight*
Proteinase K (20 mg/mL, RNA grade)	*Invitrogen 25530049*	*Engyodontium album*	*IHC & ISH*	*10mcg/mL*	*Balance at room temperature for 30 min before use*
PureLinkTM Rnase A (20 mg/ml)	*Thermo Fisher 12091021*	*Cattle*	*ISH*	*20 or 2000mcg/mL*	*Dilute in RNase buffer and incubate at 37 degree for 30min*

Supplementary Figure 1

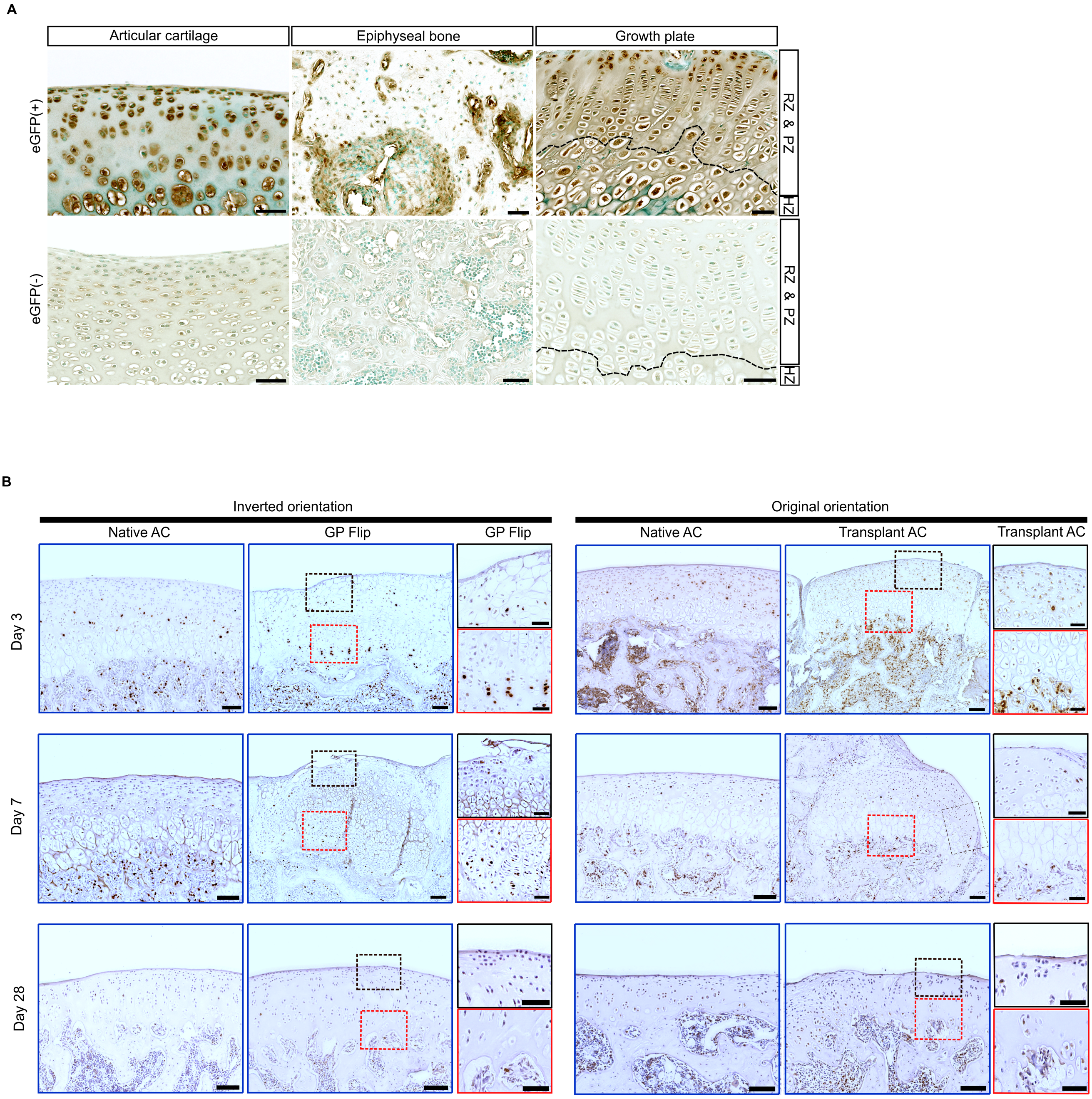

Supplementary figure 1. Cell proliferation and chondrocyte marker expression
(A) Distal femurs were collected from inbred GFP-expressing and wild type rats and used as positive and negative controls for the GFP immunohistochemistry. (B) For cell proliferation studies, transplant recipient rats received an i.p. injection of BrdU (50 mg/kg) 4 hrs before being euthanized. BrdU incorporation into cells undergoing mitosis (S-phase) was visualized by BrdU immunohistochemistry (brown coloration) displayed in low and high power. Black and red dashed squares indicate the magnified regions. Note that in the inverted growth plate cartilage transplanted to the articular surface, there were active cell proliferation at the location of the proliferative zone and interestingly also at the location of the former hypertrophic zone at postoperative day 3 and 7, whereas at day 28, proliferation had decreased and the cell proliferation pattern was more similar to articular cartilage that had been transplanted to its original location (control) and the surrounding native cartilage with a low number of BrdU labelled cells primarily located in the superficial zone. Scale bar: 50 µm (A), 100 µm in low and 50 µm in high power (B). RZ: Resting Zone, PZ: Proliferative zone, HZ: Hypertrophic Zone. The curvy dashed line roughly defines the borderline between PZ and HZ.

Supplementary Figure 2

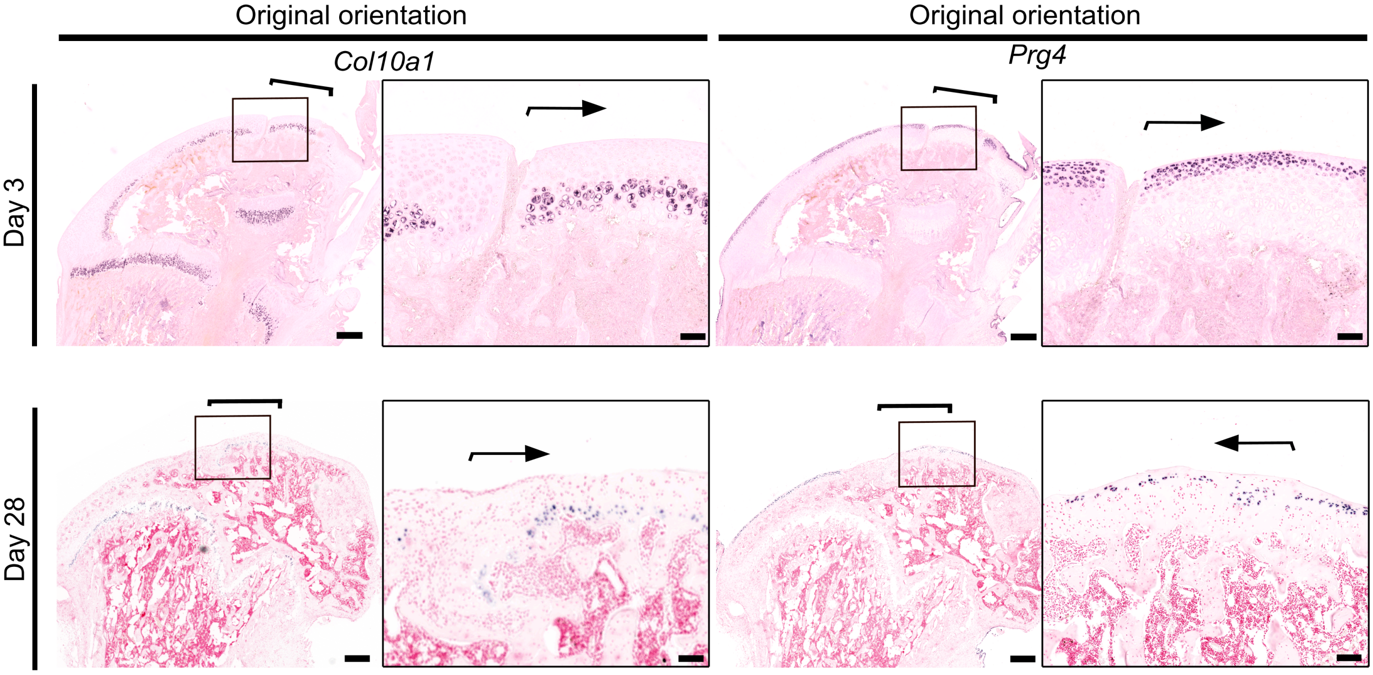

Supplementary figure 2. Localization of Col10a1 and Prg4 expression in transplants inserted in original orientation at day 3 and day 28 post surgery

Osteochondral allografts were harvested from distal femurs of inbred GFP-expressing rats and transplanted to matching sites of wild-type (GFP-negative) littermates in original orientation and localized by GFP immunohistochemistry. Hypertrophic and superficial zone chondrocyte differentiation was assessed by Col10a1 and Prg4 in situ hybridization (purple coloration) on consecutive distal femoral sections of recipient rats at postoperative day 3 and 28. Brackets and arrows delineate the location of the transplanted osteochondral allografts. Scale bar: 500 µm and 100 µm in low- and high-power images, respectively.

Supplementary Figure 3

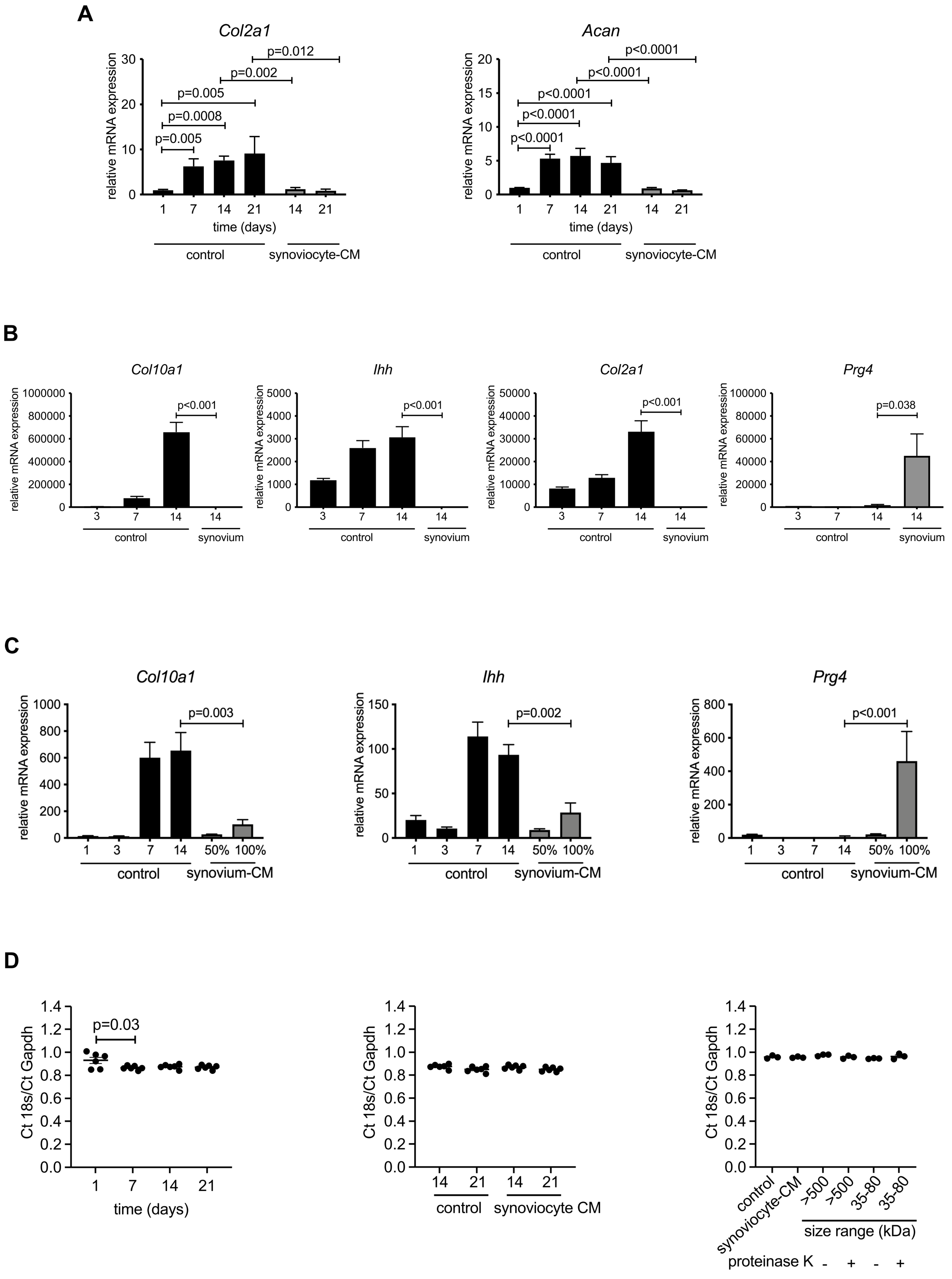

Supplementary figure 3. Differentiation of chondrocyte pellet cultured without and with synovium condition media and validation of endogenous control gene 18S rRNA.

(A) Gene expression analysis by quantitative PCR of Col2a1 and Acan in epiphyseal chondrocyte pellets cultured in chondrogenic conditions (control) and synoviocyte conditioned medium (CM). (B) Gene expression analysis by quantitative PCR of Col10a1, Ihh, Col2a1, and Prg4 in epiphyseal chondrocyte pellets cultured in chondrogenic conditions (control) over time, and with synovial tissue added to the cultures (synovium). (C) Gene expression analysis by quantitative PCR of Col10a1, Ihh and Prg4 in epiphyseal chondrocyte pellets cultured in chondrogenic conditions (control) over time, and with medium (50% or 100% conditioned media) conditioned with synovium tissue pieces (synovium-CM) at day 14. (D) To validate the endogenous control gene, expression of 18S rRNA and Gapdh were assessed by quantitative PCR in pellet cultures with culture time, without (control) and with synoviocyte-conditioned media, and in different fractions undigested or digested with proteinase K and the 18S rRNA/Gapdh cycle treshold (Ct) ratios were calculated. Except for 1 day time-point, the Ct 18S rRNA/Ct Gapdh ratios did not change between different conditions as assessed by one-way ANOVA followed by Tukey's multiple comparisons test. Thus, indicating that 18S rRNA is a suitable endogenous control gene for chondrocyte pellet culture experiments. A-C: One-way ANOVA followed by relevant pairwise comparisons assessed using Dunnett's method. Bars represent average ± SEM.

Supplementary Figure 4

A

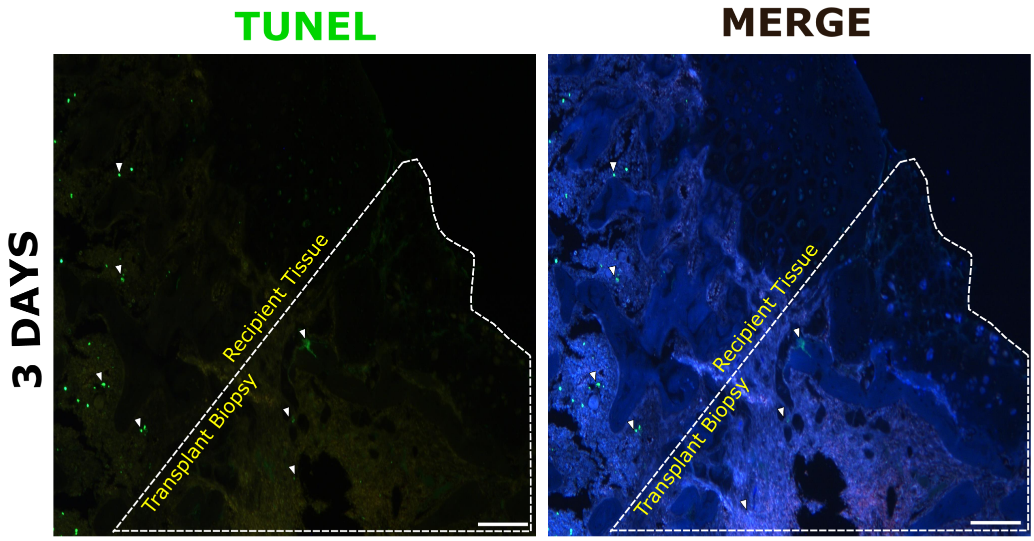

B

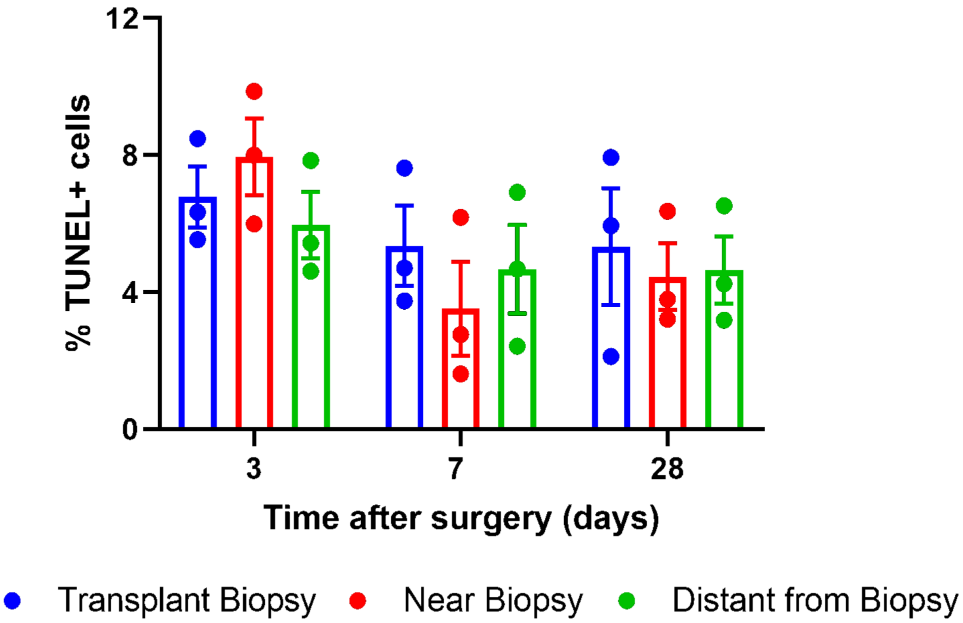

Supplementary figure 4. TUNEL assay to detect apoptotic cells in osteochondral allografts. *(A) Immunoflourescence images in a representative day 3 time-point osteochondral allograft sample (10X magnification, scale bar – 200 μm). TUNEL positive cells are displayed with an individual channel for TUNEL (green), and a merged image with DAPI (blue). The dashed lines mark the location of the osteochondral allograft. (B) Percent TUNEL positive cells at three different regions (within, near and distant from the osteochondral allograft biopsy; n=3 per each time point). There was an overall decline in the percent TUNEL positive cells with age ($P < 0.05$ by 2-way ANOVA).*
