# Supplementary material for: The synovial microenvironment suppresses chondrocyte hypertrophy and promotes articular chondrocyte differentiation
Source: NPJ Regen Med. 2022 Sep 16;7:51. doi: 10.1038/s41536-022-00247-2 (PMC9481641; doi:10.1038/s41536-022-00247-2)
Supplement: Supplementary file 2 — REPORTING SUMMARY [file 41536_2022_247_MOESM2_ESM.pdf]

nature portfolio

Corresponding author(s): Ola Nilsson

Last updated by author(s): Apr 29, 2022

Reporting Summary

Nature Portfolio wishes to improve the reproducibility of the work that we publish. This form provides structure for consistency and transparency in reporting. For further information on Nature Portfolio policies, see our Editorial Policies and the Editorial Policy Checklist.

Statistics

For all statistical analyses, confirm that the following items are present in the figure legend, table legend, main text, or Methods section.

n/a	Confirmed	
☐	☒	The exact sample size (*n*) for each experimental group/condition, given as a discrete number and unit of measurement
☐	☒	A statement on whether measurements were taken from distinct samples or whether the same sample was measured repeatedly
☐	☒	The statistical test(s) used AND whether they are one- or two-sided<br>*Only common tests should be described solely by name; describe more complex techniques in the Methods section.*
☒	☐	A description of all covariates tested
☐	☒	A description of any assumptions or corrections, such as tests of normality and adjustment for multiple comparisons
☐	☒	A full description of the statistical parameters including central tendency (e.g. means) or other basic estimates (e.g. regression coefficient) AND variation (e.g. standard deviation) or associated estimates of uncertainty (e.g. confidence intervals)
☐	☒	For null hypothesis testing, the test statistic (e.g. *F*, *t*, *r*) with confidence intervals, effect sizes, degrees of freedom and *P* value noted<br>*Give P values as exact values whenever suitable.*
☒	☐	For Bayesian analysis, information on the choice of priors and Markov chain Monte Carlo settings
☒	☐	For hierarchical and complex designs, identification of the appropriate level for tests and full reporting of outcomes
☒	☐	Estimates of effect sizes (e.g. Cohen's *d*, Pearson's *r*), indicating how they were calculated

Our web collection on statistics for biologists contains articles on many of the points above.

Software and code

Policy information about availability of computer code

Data collection	Commercial software Caseviewer v 2.3 and Aperio Imagescope v 12.4 were used for thickness and distance measurement. Caseviewer was used for necessary adjustment of image brightness and contrast. Image J was used for cell counting. Adobe Illustrator, Inkscape and Image J were used for figure preparation.
Data analysis	Graphpad was used to perform statistical analysis.

For manuscripts utilizing custom algorithms or software that are central to the research but not yet described in published literature, software must be made available to editors and reviewers. We strongly encourage code deposition in a community repository (e.g. GitHub). See the Nature Portfolio guidelines for submitting code & software for further information.

Data

Policy information about availability of data

All manuscripts must include a data availability statement. This statement should provide the following information, where applicable:

- Accession codes, unique identifiers, or web links for publicly available datasets
- A description of any restrictions on data availability
- For clinical datasets or third party data, please ensure that the statement adheres to our policy

Provide your data availability statement here.

Field-specific reporting

Please select the one below that is the best fit for your research. If you are not sure, read the appropriate sections before making your selection.

☒ Life sciences ☐ Behavioural & social sciences ☐ Ecological, evolutionary & environmental sciences

For a reference copy of the document with all sections, see nature.com/documents/nr-reporting-summary-flat.pdf

Life sciences study design

All studies must disclose on these points even when the disclosure is negative.

Sample size	we expected obvious phenotypic difference between experimental and the control group, thus the limited three animals were finally decided per time point each group considering the 3R principles.
Data exclusions	At the beginning of surgery experiments, two animals were used for protocol optimization and were not included for data report
Replication	All in vivo and in vitro experiments were replicated and successful.
Randomization	Animals with the correct genotype were randomly assigned to group (original or inverted orientation) and randomly assigned to one of 3 time-points at each surgery.
Blinding	Histomorphometric measurements were performed by one expert blinded to time-point and orientation. However, transplant morphology was often recognizable during analyses complicating blinding.

Reporting for specific materials, systems and methods

We require information from authors about some types of materials, experimental systems and methods used in many studies. Here, indicate whether each material, system or method listed is relevant to your study. If you are not sure if a list item applies to your research, read the appropriate section before selecting a response.

Materials & experimental systems

n/a	Involved in the study	
☐	☒	Antibodies
☐	☒	Eukaryotic cell lines
☒	☐	Palaeontology and archaeology
☐	☒	Animals and other organisms
☒	☐	Human research participants
☒	☐	Clinical data
☒	☐	Dual use research of concern

Methods

n/a	Involved in the study	
☒	☐	ChIP-seq
☒	☐	Flow cytometry
☒	☐	MRI-based neuroimaging

Antibodies

Antibodies used	Anti-GFP primary antibody Abcam, ab290,<br>Anti-BrdU primary antibody Abcam, ab152095,<br>Biotinylated goat anti-rabbit IgG secondary antibody Vector Labs PK-4001,<br>Anti-Digoxigenin-AP, Fab fragments Roche, 11093274910
Validation	ab290 was a Rabbit raised polyclonal antibody against to GFP. Anti-GFP antibody (ab290) is a highly versatile antibody that gives a stronger signal than other anti-GFP antibodies available. On Western blot the antibody detects the GFP fraction from cell extracts expressing recombinant GFP fusion proteins and has also been shown to be useful on mouse sections fixed with formalin. In Immunocytochemistry, the antibody gives a very good signal on recombinant YES-GFP chimeras expressed in COS cells (McCabe et al. 1999 and figure below). It is routinely used in Immunoprecipitation (IP) and IP-Western protocols and has been used successfully in HRP Immunohistochemistry at 1:200 on whole-mount mouse embryos.GFP antibody is reactive against all variants of Aequorea victoria GFP such as S65T-GFP, RS-GFP, YFP, CFP, RFP and EGFP.Targeting is species independent. IHC with paraffin sections was validated with antibody dilution of 1/500 - 1/1000. Perform heat mediated antigen retrieval via the microwave method before commencing with IHC staining protocol. Positive and negative control images were provided as supplemental materials in this study.<br><br>ab152095 is a Rabbit raised polyclonal antibody agains to BrdU. Targeting is species independent and targeting specificity was validated by Abcam in applications of BrdU immunofluorescence and flowcytometry with Hela cells, immunohistochemistry for mouse intestine. Immunohistochemistry (Formalin/PFA-fixed paraffin-embedded sections) of mouse BrdU treated intestine using ab152095 diluted at 1/500.

Eukaryotic cell lines

Policy information about cell lines

Cell line source(s)	The commercial synoviocyte cell line HIG-82 CRL-1832 is from ATCC
Authentication	As noted by ATCC, this cell line was derived from the intrarticular soft tissue from the knee joint of a young female rabbit. This cell line has retained many of the features of normal rabbit synoviocytes including production of cytokines that activate primary cultures of normal chondrocytes. These cells can be activated with PMA or IL-1, and can phagocytose latex beads [PubMed: 2846503].
Mycoplasma contamination	It was confirmed that our cell line is negative to Mycoplasma contamination
Commonly misidentified lines (See ICLAC register)	NA

Animals and other organisms

Policy information about studies involving animals; ARRIVE guidelines recommended for reporting animal research

Laboratory animals	Rat species Lew-Tg(CAG-EGFP)YsRrrc from RRRC (https://www.rrrc.us/Strain/?x=296&log=yes). Animals were used regardless of gender and the age is four week at the operation day.
Wild animals	NA
Field-collected samples	NA
Ethics oversight	All animal procedures were approved by the Animal Care and Use Committee, National Institute of Child Health and Human Development (Animal Study Proposal number: 13-087) and the regional animal ethics committee in Stockholm (Permit no. N248/15, N118/16, and 15635-2017).

Note that full information on the approval of the study protocol must also be provided in the manuscript.
